# Sports-Related Health Problems in Para-Sports: A Systematic Review With Quality Assessment

**DOI:** 10.1177/19417381231178534

**Published:** 2023-06-19

**Authors:** Sietske C.M. Luijten, Leonie M. te Loo, Joske Nauta, Thomas W.J. Janssen, Jasmijn F.M. Holla, René H.J. Otten, Ingrid Vriend, Evert Verhagen

**Affiliations:** Amsterdam Collaboration on Health & Safety in Sports, Amsterdam, Department of Public and Occupational Health, Amsterdam Movement Sciences, Amsterdam UMC, Vrije Universiteit Amsterdam, Amsterdam, the Netherlands; Faculty of Health, Sports and Social Work, Inholland University of Applied Sciences, Haarlem, The Netherlands; Amsterdam Collaboration on Health & Safety in Sports, Amsterdam, Department of Public and Occupational Health, Amsterdam Movement Sciences, Amsterdam UMC, Vrije Universiteit Amsterdam, Amsterdam, the Netherlands; Department of Human Movement Sciences, Faculty of Behavioural and Movement Sciences, Vrije Universiteit Amsterdam, Amsterdam Movement Sciences, Amsterdam, the Netherlands, and Amsterdam Rehabilitation Research Centre, Reade, Amsterdam, The Netherlands Center for Adapted Sports Amsterdam, Amsterdam Institute of Sport Science, Amsterdam, the Netherlands; Faculty of Health, Sports and Social Work, Inholland University of Applied Sciences, Haarlem, The Netherlands, Amsterdam Rehabilitation Research Centre, Reade, Amsterdam, The Netherlands, and Center for Adapted Sports Amsterdam, Amsterdam Institute of Sport Science, Amsterdam, the Netherlands; Medical Information Specialist, Medical Library, Vrije Universiteit Amsterdam, Amsterdam, The Netherlands);; Amsterdam Collaboration on Health and Safety in Sports, Amsterdam, Department of Public and Occupational Health, Amsterdam Movement Sciences, Amsterdam UMC, Vrije Universiteit Amsterdam, Amsterdam, the Netherlands; Amsterdam Collaboration on Health and Safety in Sports, Amsterdam, Department of Public and Occupational Health, Amsterdam Movement Sciences, Amsterdam UMC, Vrije Universiteit Amsterdam, Amsterdam, the Netherlands

**Keywords:** athletic injuries, epidemiology, sports for persons with disabilities, systematic review

## Abstract

**Context:**

Participation in sports is associated with a risk of sports-related health problems. For athletes with an impairment, sports-related health problems further burden an already restricted lifestyle, underlining the importance of prevention strategies in para-sports.

**Objective:**

To provide a comprehensive overview with quality assessment of the literature on sports-related health problems, their etiology, and available preventive measures in para-sports following the steps of the Sequence of Prevention.

**Data Sources:**

A literature search (in PubMed, Embase, SPORTDiscus, CINAHL and the Cochrane Library) was performed up to December 8, 2021, in collaboration with a medical information specialist.

**Study Selection:**

The search yielded 3006 articles, of which 64 met all inclusion criteria.

**Study Design:**

Systematic review with quality assessment.

**Level of Evidence:**

Level 3.

**Data Extraction:**

Two independent researchers carried out the screening process and quality assessment. One researcher extracted data, and the Sequence of Prevention categorized evidence.

**Results:**

A total of 64 studies were included, of which 61 reported on the magnitude and risk factors of sports-related health problems, while only 3 reported on the effectiveness of preventive measures. Of these, 30 studies were of high quality. Most studies (84%) included elite-level athletes. The reported injury incidence varied widely between sports (0-91 per 1000 athlete days) and impairment categories (1-50 per 1000 athlete days). The same applies to illness incidence with regard to different sports (3-49 per 1000 athlete days) and impairment categories (6-14 per 1000 athlete days).

**Conclusion:**

This review shows the current vast range of reported sport-related health problems in para-sports. There is limited evidence concerning the severity of these sports-related health problems and inconclusive evidence on the risk factors. Lastly, the evidence regarding the development and effectiveness of preventive measures for para-athletes is sparse.

Physical activity improves health, social life, and quality of life in nondisabled and disabled people.^7,44,46,82^ The World Health Organization recommends that people with impairments do aerobic and multicomponent physical activity that emphasizes functional balance and strength to maintain an active lifestyle.^8^ Sports can help to get enough exercise.

Sports have many benefits but also increase the risk of sports-related injuries and illnesses (eg, respiratory infections or psychological health complaints). Health issues can make daily life harder for people with physical disabilities, so should be prevented.^82,90^ Due to their impairments or mobility aids, disabled athletes may have different health issues than nondisabled athletes.^29,30^

Comprehensive reviews of injury incidence in athletes with physical impairments have improved understanding.^67,81,87,90^ However, injury and illness incidence by sport and impairment category, etiology, and prevention strategies are unavailable. Given the high incidence of injuries in para-sports,^67^ an overview of injury and illness numbers divided by sport and impairment category, etiology, and preventive strategies could help explain sports-related health problem mechanisms. It would also aid prevention intervention research.

Thus, this review provides a comprehensive overview and quality assessment of the literature on sports-related health problems, their etiology, preventive measures, and their efficacy in para-sports following the Sequence of Prevention.^83^

## Methods

This study was conducted following Preferred Reporting Items for Systematic Reviews and Meta-Analyses (PRISMA) guidelines,^65^ and was registered in PROSPERO.^84^

### Information Sources and Search

To identify all relevant publications on sports-related health problems in athletes with physical impairments, we performed systematic searches in PubMed, EMBASE, SPORTDiscus, and CINAHL (via EBSCO) Cochrane Library (via Wiley) from inception to December 8, 2021. Search terms included controlled terms (like MeSH in PubMed and Emtree in Embase) and free-text terms. In the Cochrane Library, we used free-text terms only. In addition, search terms expressing “sports injuries” were combined with search terms comprising “individuals with physical disabilities.” The full search strategies for all databases can be found in Appendix 1 (available in the online version of this article).

### Eligibility Criteria

We established our inclusion and exclusion criteria with mutual agreement:

- Study aim: the included studies should provide information regarding ≥1 of the steps of the sequence of prevention.
- Study design: we included only observational, descriptive, and prospective cohort studies and clinical trials. To minimize errors that often occur during data recall, retrospective studies were excluded.^14^
- Participants: we considered studies only if they included athletes with a physical (motor), visual, or hearing impairment. Studies including only athletes with intellectual impairments were excluded.
- Type of sports: we considered all sports and made no distinction between the level of sports performance, impairment category, age, or sex.
- Sports-related health problem definition: we accepted all definitions used in the respective studies. To provide a clear overview of sports-related health problems, they are divided into injuries and illnesses following the definitions reported in the Para-sport translation of the International Olympic Committee (IOC) consensus statement on recording and reporting of data for injury and illness in sport.^18^
- Language: only publications in English were included.

### Selection Procedure

The selection process was performed in 3 phases. In the first phase, all titles were screened by 1 author (for pragmatic reasons), and exclusions were made conservatively according to the predetermined criteria. Two authors separately screened all remaining abstracts, followed by a consensus meeting to discuss different ratings. A third author was consulted when a consensus could not be reached. This was done on 3 occasions. The third phase consisted of both authors reading the full texts to determine whether the article met the predetermined criteria, followed by the same discussion procedure until a consensus was reached and a definite selection of articles was made. Finally, the reference lists of the included articles were scanned to ensure no article was missed.

### Data Extraction

The data extraction, performed by 1 author, was based on the steps of the Sequence of Prevention.^34,83^

Related to the first step, various data were extracted: authors, publication year, title, study design, sports-related health problem definition, follow-up duration, sport, impairment type, sports-related health problem numbers, nature of sports-related health problems, and severity. The data were then pooled per sport and impairment. Sports categories were based on individual sports and a combination of winter or summer Paralympic sports (Table 1). Impairment categories were based on the categories reported in the included literature: visual impairment, limb deficiency, spinal cord injury, central neurologic injury, hearing impairment, and other, as well as disabled sports (Table 2).

**Table 1.** Summary of reported injury rates, injury severity, and injury onset in para-sports categorized by sports type

Sport	Injury Prevalence	Injury Incidence	Severe Injuries	Severity	Sudden Onset	Gradual Onset
Alpine skiing	12%-37%^19,24,88,89^	23-4^19,24 b^	-	-	45%-79%^88,89^	8%-52%^88,89^
Archery	11%-60%^20,68,92^	8-68^20,54,92,94 b^	-	-	33%^92^	47%^92^
Basketball	8%-79%^16,20,47,61,68,72,79,92^	0-72^20,47,53,54,92,94 b^	33^53^	6^47 t^ 2^64 x^	40%-65%^47,53,92^	23%-52%^47,53,92^
Boccia	6%-11%^20,92^	8-12^20,92 b^	-	-	91%^92^	9%^92^
Canoe	12%^20^	1020^b^	-	-	-	-
Cycling (track and road)	9%-17%^20,68,92^	7-58^20,54,92 b^	-	-	71%-75%^92^	17%-25%^92^
Equestrian	10%-13%^20,92^	7-73^20,54,92 b^	-	-	56%^92^	22%^92^
Fencing	18%-71%^20,68,92^	16-91^20,54,92 b^ 4^13 c^	4^13 l^	-	42%-62%^13,92^	39%-58%^13,92^
Football 5%-a%-side	24%-63%^20,71,86,92^	22-23^20,86,92 b^ 0.1^73 d^ 0.87^71 i^	-	33^20 s^	55%-80%^73,86,92^	20%-23%^73,86,92^
Football 7%-a%-side	15%-19%^20,86,92^	10-15^20,86,92 b^ 0.05^32 h^	5^86 k^	26^20 s^	71%-73%^86,92^	7%^86,92^
Goalball	7%-27%^20,92^	6-20^20,92 b^	-	-	77%^92^	13%^92^
Judo	17%-80%^20,68,92^	16-55^20,54,92 b^ 69^40 j^	-	16^20 s^	64%^92^	20%^92^
Nordic skiing	2%-19%^19,24,88,89^	8-14^19,24 b^	-	-	33%-46%^88,89^	54%-66%^88,89^
Para snowboard	33%^19^	41^19 b^	-	-	-	-
Power lifting	16%-75%^20,63,68,91,92^	11-91^20,54,63,92 b^ 33.3^91 e^	-	-	13%-18%^63,91,92^	61%-64%^63,91,92^
Rowing	6%-9%^20,92^	4-80^20,53,92 b^	-	-	60%^92^	40%^92^
Sailing	6%-11%^20,92^	4-36^20,53,92 b^	-	-	50%^92^	25%^92^
Shooting	3%-55%^20,68,92^	2-57^20,54,92 b^	-	-	100%^92^	-
Sledge hockey	14%-34%^19,24,88,89^	23-27^24,25 b^	-	-	40%-83%^88,89^	17%-60%^88,89^
Softball	-	9.8^94 b^	-	-	-	-
Swimming	9%-69%^4,20,68,92^	8-65^20,54,92 d^0.3^74 b^	-	-	20%-47%^74,92^	37%-80%^74,92^
Table tennis	11%-69%^20,68,92^	9-46^20,54,92 b^	-	-	47%^92^	45%^92^
Tennis	14%-75%^20,51,68,92^	11-68^20,54,92 b^	-	-	37%^92^	47%^92^
Track and field	12%-80%^5,20,68,75,92^	10-61^5,20,54,92 b^ 2^75 g^	6.7^5 p^ 4.1^5 q^	-	18%-50%^5,75,92^	29%-82%^5,75,92^
Triathlon	12%^20^	10^20 b^	-	-	-	-
Volleyball	13%-90%^20,68,92^	11-46^20,54,92,94 b^	-	-	65%^92^	22%^92^
Weightlifting	50%^68^	^-^	-	-	-	-
Wheelchair curling	8%-18%^19,24,88^	7-17^19,24 b^	-	-	0%^88^	100%^88^
Wheelchair rugby	17%-25%^20,60,92^	15-16^20,92 b^ 0.3^4 h^	-	-	61%^92^	17%^92^
Combined Paralympic sports	4%-8%^6,12,59^ 20%^78 a^	7-9^29,32 c^ 2^31 f^ 8^78 i^	31%-52%^32,55 m^ 29-31^32,55 n^ 19-38^32,55 o^	17^32 w^ 14^78 v^	20%-68%^31,33,78^ 5%^78 y^	21%-80%^31,33,78^ 15%^78 y^
Combined Summer Paralympic sports	9%-82%^9,55,68^ 18%^45,78 a^	8-64^20,22,39,45,54,92 b^ 3^76 g^ 1-7^11,29,55 c^	20%-25%^20,54^	2-3^45 u^	7%-67%^20,22,54,62,76,92^ 3-13^39,45 z^ 2^11 aa^ 4%^78 y^	30%-93%^20,22,54,62,76,92^ 5-17^39,45 z^ 2^11 aa^ 15%^78 y^
Combined Winter Paralympic sports	9%-24%^19,55,88,89^ 23%^78 a^	21-27^19,24 b^ 0.4-7^29,55 c^	12%-27%^19,24,88,89^	-	41%-77%^19,24,88,89^ 7%^78 y^	15%-58%^19,24,88,89^ 16%^78 y^
Combined wheelchair sports	17%-48%^10,17,35,36^	-	27^32 m^,12^32 n^,8^32 o^	-	-	-

IR, incidence rate.

a Mean weekly prevalence.

b IR per 1000 athlete days.

c IR per 1000 hours of exposure.

d IR per match.

e IR per 1000 athlete-competition days.

f IR per 100 hours.

g Per participating athlete.

h Per athlete training day.

i Per athlete year.

j IR per 1000 athlete exposures.

k Time loss >1 day.

l Time loss >22 days.

m Time loss 1-7 days.

n Time loss 8-21 days.

o Time loss >21 days.

p IR of time-loss injuries in ambulant athletes.

q IR of time-loss injuries in wheelchair athletes.

r

s Time lost per 1000 athlete days.

t Time loss injuries per 100 players.

u Time loss in days.

v Time loss per year.

w Group mean number of days lost.

x Mean duration of shoulder pain in years.

y average weekly prevalence.

z IR per 1000 athlete days.

aa IR per 1000 hours of exposure.

**Table 2.** Summary of reported injury rates, injury severity, and injury onset in para-sports categorized by impairment category

Category	Injury Prevalence	Injury Incidence	Severe Injuries	Severity	Sudden Onset	Gradual Onset
Visual impairment	63%-85%^71,73-76^ 14-31^5,29,40,55,86 c^ 9%-22%^19,20,33,54 a^	1-33^5,39,88,94 d^ 2^74,75 g^ 0.1^73 f^ 1-10^11,29,55 e^ 69^40 i^ 0.9^71 h^	9^32 k^,6^32 l^,1^32 m^	15^40 n^	18%-100%^39,73-76,86^ 3.5^11 o^	0-82%^39,73-76,86^ 35^11 o^
Musculoskeletal	0-38%^1,72^ 22%^78 b^	-	-	-	-	-
Limb deficiency	30%-54%^19,20,54 a^ 9-26^5,29 c^	4-40^5,39 d^ 0.05^52 j^ 3-8^11,29 e^	-	-	55%-100%^39^ 1.6^11 o^	0-45%^39^ 1.8^11 o^
Impaired passive range of movement	-	-	-	-	-	-
Short stature	35^5 c^ 1%-2%^20,33 a^	50^5 d^	-	-	-	-
Neurological	18%^78 b^	-	-	-	-	-
Spinal cord injury	25%-69%^10,60,61^ 20%-34%^19,20,54 a^ 11-27^29,55 c^	4-12^39,45 d^ 0.3^4 j^ 1-8^11,29,55 e^	-	-	36%-48%^39,45^ 1.6^11 o^	52%-64%^39,45^ 1.2^11 o^
Central neurologic injury	86%^72^ 10-15^29,86 c^ 7%-16%^19,20 a^	10^86 d^ 3-5^11,29 e^	-	-	93%^86^ 1.8^11 o^	7%^86^ 1.4^11 o^
Brain disorder	9-15^5 c^ 10%-24%^33,54 a^	4-19^5,39 d^	22^32 k^,15^32 l^,7^32 m^	-	17%-36%^39^	64%-83%^39^
Hearing impairment	4%^6^	-	-	-	-	-
Other (often referred to as “les autres”)	0-8%^19,20 a^ 6^29 c^	2-4^39 d^ 6^45 d,p^ 2-3^11,29 e^	-	-	14%-100%^39,45 p^ 1.2^11 o^	0-86%^39,45 p^ 1.2^11 o^

IR, incidence rate.

a Percentage of total injuries in this impairment category.

b Average weekly prevalence.

c Incidence proportion per 100 athletes.

d IR per 1000 athlete days.

e IR per 1000 hours of exposure.

f IR per match.

g Per participating athlete.

h Per athlete year.

i IR per 1000 athlete exposures.

j IR per athlete per training day.

k Minor injury of 0-7 days lost.

l Moderate injury of 8-21 days lost.

m Major injury of >22 days lost.

n IR injuries resulting in inability to continue.

o IR per 1000 hours of exposure.

p Non-spinal-cord injury.

The results for the second step of the Sequence of Prevention, etiology, are presented according to the Injury Causation model.^3,58^ This model considers the differences between intrinsic and extrinsic and modifiable and nonmodifiable factors. While the interpretation of this model is quite straightforward, some risk factors can be categorized as modifiable and nonmodifiable. This applies, for example, to the risk factor “sports experience” since experience can change over time. We classified all factors with injury prevention interventions in mind. Because an intervention cannot change an athlete’s sports experience at the time of inclusion, it is classified as nonmodifiable. All risk factors are classified accordingly.

The final 2 steps of the model, ie, the development of sports-related health problem prevention interventions and their effectiveness, were combined. In addition, information regarding sport, follow-up duration, sample size, intervention, outcome measures, and results were extracted.

### Quality Assessment

Two authors independently assessed the methodological quality of each included study. The quality assessment of the studies reporting on the first 2 steps of the model was based on the 10-item tool by Hoy et al^48,49^ (Appendix 5, available online). Studies with 8 to 10 points were considered “high quality”, with 5 to 7 points “medium quality”, and with ≤4 points “low quality.” To assess the quality of the clinical trials, we employed the Cochrane Risk of Bias assessment tool.^15^ Summary assessment per study was determined as follows: only when all key domains scored “low risk of bias” would the summary score be “low risk of bias”; if ≥1 key domains scored “unclear risk of bias,” the summary score was “unclear risk of bias,” unless there were ≥1 key domains that scored “high risk of bias,” in which case the summary score would be “high risk of bias.” Any conflicts between both assessors were discussed, and a consensus was reached.

## Results

**Figure 1.**
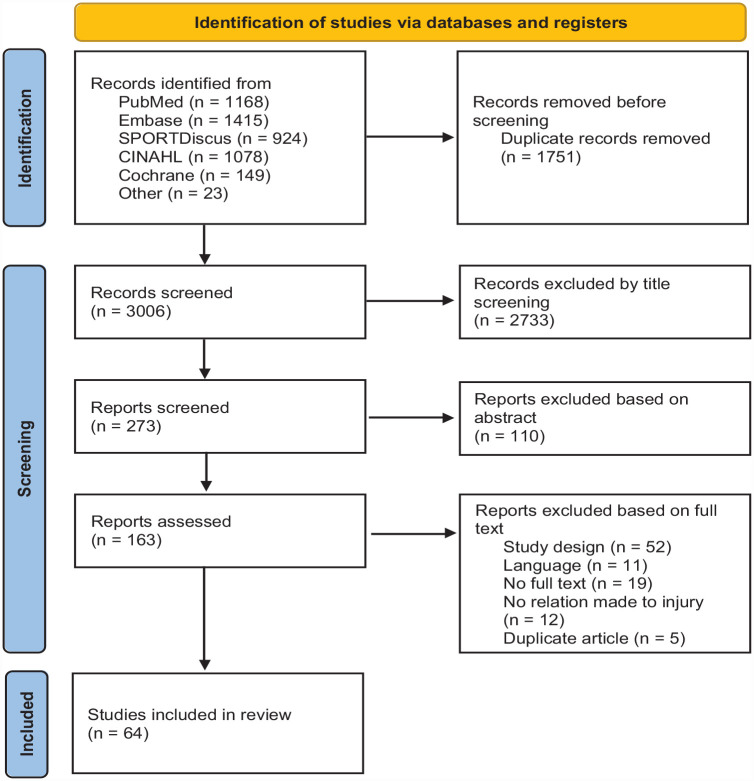
Overview of the systematic search and selection procedure.

Our systematic search yielded 3006 studies, of which we included 62 studies. Two additional studies were included after reviewing the reference lists of included studies. The study identification procedure is visualized in Figure 1.

### Identification of Studies

Most studies were descriptive, observational, or prospective cohort designs (73%), focusing predominantly on the first and second steps of the Sequence of Prevention. The cross-sectional studies (23%) focused mostly on step 2, while the clinical trials (4%) focused on the third and fourth steps of the Sequence of Prevention. Follow-up duration of the included prospective studies varied between 7 days and 9 years. The sample size ranged from 8 to 4378 participants, of which 42% were male, 22% female, and not specified for 36% of participants. Most studies included athletes of an elite level (84%), and only 2 studies included athletes of a recreational level. The level of play of the participants in the remaining studies was not mentioned. An overview of included sports and impairment types is provided in Tables 1 and 2. A summary of the data extraction is presented in Appendix 2, available online. A total of 30 studies concentrating on the first 2 steps were of high quality, 16 of medium level, and 15 of low quality. All 3 studies focusing on steps 3 and 4 of the Sequence of Prevention had a high risk of bias. An overview of the quality assessment is in Appendices 5 and 6, available online.

### Step 1: Injury Rates in Para-Sports

Of the 52 studies reporting injury rates in para-sports, 31% reported rates for 1 sport, 32% reported injury rates for multiple sports, and 37% provided a combined overall rate for multiple sports. For the impairment categories, 27% reported 1 category, 23% gave a breakdown of the rates between various categories, and 52% did not mention the impairments or did not give a breakdown per category.

Tables 1 and 2 present the reported prevalence, incidence, injury severity, and percentage of sudden and gradual onset injuries of the included studies between and within various sports and impairment categories. Of all studies that reported injury incidence, 47% expressed rates as a ratio between injuries and sports exposure per 1000 athlete days while the remaining studies used different units. Most studies reported the prevalence and incidence of injury. In contrast, 7 studies reported severe injuries or injury severity per sport, and 10 studies in combined Paralympic sports.

### Step 1: Illness Rates in Para-Sports

Of the 64 studies, 11 reported on illness prevalence and incidence. A summary of the data extraction is presented in Appendix 3, available online. Of these 11 studies, 2 reported illness prevalence and incidence rates in athletes classified per sport, and 9 in combined Paralympic sports (Table 3). Overall, illness severity was reported by 7 studies. Furthermore, 4 studies reported illness prevalence per impairment category (Table 4). Incidence rates and severity per impairment category were reported in 1 study.

**Table 3.** Summary of reported illness rates and illness severity in para-sports categorized by sports type

Sport	Illness Prevalence	Illness Incidence	Severity
Alpine skiing	19%^25^	19 (14-27.0)^25 b^	-
Archery	11%^23^	9 (5-15)^23 b^	-
Basketball	15%^23^	13 (9-17)^23 b^	57^53^
Boccia	16%^23^	12 (8-20)^23 b^	-
Canoe	17%^23^	14 (7-26)^23 b^	-
Cycling (track and road)	13%^23^	11 (7-15)^23 b^	-
Equestrian	11%^23^	9 (5-17)^23 b^	-
Fencing	15%^23^	15 (9-25)^23 b^	-
Football 5-a-side	6%^23^	4 (2-11)^23 b^	-
Football 7-a-side	5%^23^	3 (1-8)^23 b^	-
Goalball	8%^23^	6 (3-11)^23 b^	-
Judo	5%^23^	4 (2-8)^23 b^	-
Nordic skiing	16%^25^	17 (11-26)^25 b^	-
Para snowboard	-	-	-
Powerlifting	10%^23^	8 (5-13)^23 b^	-
Rowing	14%^23^	10 (6-17)^23 b^	-
Sailing	13%^23^	11 (6-20)^23 b^	-
Shooting	17%^23^	12 (8-18)^23 b^	-
Sledge hockey	14%^25^	19 (13-30)^25 b^	-
Swimming	15%^23^	13 (10-16)^23 b^	-
Table tennis	12%^23^	9 (7-13)^23 b^	-
Tennis	7%^23^	8 (4-14)^23 b^	-
Track and field	13%^23^	10 (9-12)^23 b^	-
Triathlon	7%^23^	5 (12-13)^23 b^	-
Volleyball	10%^23^	8 (5-13)^23 b^	-
Weightlifting	-	-	-
Wheelchair curling	24%^25^	20 (10-40)^25 b^	-
Wheelchair rugby	16%^23^	13 (8-21)^23 b^	-
Paralympic sports	19%^78 a^	2^31 d^ 9^29 c^	18^78 f^
Summer Paralympic sports	11-19%^45,78 a^	8-49^21-23,39,45b^ 0.6^11 e^	10-16%^21,23 g^ 3^31 h^ 3^45 i^
Winter Paralympic sports	20%^78 a^	19^25 b^	21^25 g^
Wheelchair sports	-	-	-

IR, incidence rate.

a Mean weekly prevalence.

b IR per 1000 athlete days.

c IR per 1000 hours of exposure.

d IR per 100 hours.

e IR per 100 athlete days.

f days lost per athlete per year.

g Time loss of >1 day.

h Time loss in training sessions.

i Time loss in days.

**Table 4.** Summary of reported illness rates in para-sports categorized by impairment category

Category	Illness Prevalence	Illness Incidence	Severity
Visual impairment	13-21%^21,23 a^	0.6 (0.2-1.2)^11 b^	-
Musculosketetal	-	-	-
Limb deficiency	24-27%^21,23 a^	0.6 (0.4-0.9)^11 b^	-
Impaired passive range of movement	-	-	-
Short stature	2%^21,23 a^	-	-
Neurological	-	-	-
Spinal cord injury	30-31%^21,23 a^	0.5 (0.3-0.9)^11 b^ 10 (8-14)^45 c^	2.8^45^
Central neurologic injury	15%^23 a^	0.6 (0.4-0.9)^11 b^	-
Brain disorder	10%^21 a^	-	-
Hearing impairment	-	-	-
Other (often referred to as “les autres”)	2-10%^21,23 a^	0.3 (0.01-1.8)^11 b^ 8 (6-10)^45 c,d^	3.5^45 d^

IR, incidence rate.

a Percentage of total illnesses per impairment category.

b IR per 100 athlete days.

c IR per 1000 athlete days.

d Non-spinal cord injury.

### Step 2: Etiology of Health Problems

A total of 51 studies reported on the etiology of sports-related health problems. The data are presented according to the classified risk factors defined in the Injury Causation Model,^3,58^ and their relations with injury and illness occurrence (Table 5).

**Table 5.** Reported risk factors for sports-related health problems in para-sports

Category Risk Factor	Relation With Sport-Related Health Problem Occurrence^a^		
	+	-	0^b^
Reported intrinsic modifiable risk factors			
Physical fitness	-	-	-
Muscle strength	10,36	-	62
Range of motion	-	62,68	36,43
Body composition	-	-	-
Body mass	-	92	41,63,65
Posture	-	-	43
Psychological	-	-	-
Coping	-	-	33
Aggressiveness	-	-	4,53
Overtraining and stress	-	-	30
Reported intrinsic nonmodifiable risk factors. For the variable sex, a “+” relation means more reported injuries in men, and a “-” means more reported injuries in women			
Impairment related	UTD	UTD	UTD
Sex	**48**^c^,**5**,**80**,82, **30**	**48**^c^,**23**,**30**,**56**,**80**	41,78,90,**19**,**20**,**24**,**55**,**65**,**76**,**77**,**89**,**92**,**93**, **25**
Age	17,37,62,82,**24**,**65**, **23**, **25**	**20**,**46**	43,52,68,**5**,**19**,**55**,**87**,**92**,**93**
Sports experience	37,82	-	52,61,63,74,**53**
Wheelchair use	16,17,63,74,**30**	-	2,61,56
Previous injury	**30**	-	-
Limb dominance	-	-	10,61
Reported extrinsic modifiable risk factors. For ‘training load’ a ‘+’ relation is defined as more injuries with more training load. For ‘equipment’ a ‘+’ relation is defined as fewer injuries with proper use of equipment			
Training load	62	**30**	43,44,**56**
Equipment	-	-	44,90,**53**
Reported extrinsic nonmodifiable risk factors. For ‘competition phase’, a ‘+’ relation is defined as more injuries during the competition phase in relation to precompetition or training. For ‘player position’, a ‘+’ relation is defined as any reported relation between player position and injuries			
Type of sports	UTD	UTD	UTD
Competition/training phase	**5**,**13**,**48**	**20**,**93**	43,44,**19**,**55**,**87**, **22**
Player position	**53**		**4**,**87**
Weather/season		**46**, **46**	**11**

UTD, unable to determine.

a Definition of a ‘+’ relation is the more of the variable, the fewer injuries. The ‘- ‘relations is the less of the variable, the more injuries. Nonunderlined references concern injury etiology; underlined references concern illness etiology; bold text indicates high-quality studies (quality assessment ≥8).

b No significant relation reported.

c More contact injuries for men and more noncontact injuries for women.

A total of 34 studies investigated the relationship between impairment and sports-related health problem occurrence,^5,6,9,10,13,19-21,29,31,36,39,40,42-44,50,52-55,57,60-62,66,72,74-76,78,80,85^ and 18 studies investigated the relationship between the type of sports and sports-related health problem occurrence.^2,5,6,19,20,23-25,29,50,54,55,59,74,78,88,89,92^ Due to the large variety in the respective type of impairment and type of sports in the included studies, it was impossible to define any single representative relation between these risk factors and health problem occurrence (Appendix 4, available online). Regarding injury etiology, the other reported risk factors show inconclusive results. Regarding illness etiology, 2 studies found a relation between higher age and greater illness occurrence. Furthermore, only 1 study reported the relationship between impairment and type of sport and illness occurrence.^29^

### Steps 3 and 4: Preventive Measures

Three studies with a high risk of bias reported on the effect of an injury prevention intervention aimed at shoulder complications in wheelchair athletes (Table 6).^29,56,93^ More detailed information on the risk of bias assessment can be found in Appendix 6, available online. All 3 studies reported perceived shoulder function, stability and range of motion, but the results were inconclusive.^38,56,93^

**Table 6.** Intervention studies on preventive measures to reduce health problems in para sports

Study	Sample Size	Intervention	Outcome Measures	Results	Risk of Bias
Garcia-Gomez et al^38^	36 wheelchair basketball players	Home-based exercise program (10 weeks), resisting and stretching exercise program and general recommendations	Shoulder pain (SPI-WB) ROM	No difference between the intervention group and control group in shoulder pain and ROM. Shoulder pain did not increase in participants that had shoulder pain at baseline. ROM-extension decreased, in the intervention group more (10%) than in the control group (5%)	High
Wilroy et al^93^	7 wheelchair basketball players	Strengthening exercises with resistive bands and stretching exercises (6 weeks)	ROM (inclinometer), Strength (handheld dynamometer)	ROM IR and ER increased on the dominant side. No significant increase in strength (IR, ER, and scapular retractor strength)	High
Maarouf et al^57^	24 wheelchair basketball athletes with spinal cord injury	Scapular stability based corrective exercises (8 weeks)	Scapula stability	The results showed a significant improvement in scapulohumeral rhythm ratio after nonpreferred hand training at 90° and 135° shoulder abduction angles	High

DASH, disabilities of the arm, shoulder and hand; ER, external rotation; IR, internal rotation; ROM, range of motion; SPI-WB, shoulder pain index for wheelchair basketball players; SRQ, shoulder rating questionnaire; WUSPI, wheelchair users shoulder pain index.

## Discussion

Our review aimed to provide an overview of the literature on the magnitude of health problems, their etiology, and preventive measures in para-sports. We identified 64 studies with heterogeneous research objectives, targeting various sports and impairment categories, making a useful comparison impossible. When studies did focus on the same outcome measure, the results were usually inconclusive. Of the 61 studies reporting on health problem magnitude and etiology, 49% were of high quality. All 3 clinical trials had a high risk of bias.

### Health Problem Rates in Para-Sports

When evaluating the reported injuries, specific results stand out. There is a wide variety in injury prevalence and incidence, and there is limited evidence on both severity of injury and type of injury (sudden or gradual onset). Regarding sports-related illnesses in para-sports, 11 studies reported prevalence and incidence; only 1 of these studies included illness severity as an outcome.

The results show a wide variety in injury prevalence and incidence per sport and impairment category, which may be due to various injury definitions used.^20,81,94^ Furthermore, almost 36% of the included studies did not define injury. Second, heterogeneity in methodologies may explain the wide variety in the reported magnitude of health problems, limiting comparison between studies. This is also visible in the calculation methods of injury prevalence and incidence. Tables 1 and 2 indicate the variation between the reported prevalence and incidence numbers, with >8 different ways to calculate injury incidence and >3 different ways to calculate prevalence. A recent consensus statement addressed both problems.^18^ When considering the comparability of various datasets, this research field could benefit from adhering to the latest consensus statement on reporting epidemiological data.

The severity of health problems in para-sports needs to be explored further: only 16 studies reported on injury severity and 1 on illness severity, and all use different definitions of severity.^20,32,85^ The consensus of included studies on severity definition is based on time lost from sports participation. A disadvantage of this narrow interpretation is that the impact on daily functioning is not taken into account. Because sports injuries can further limit daily activities in athletes with disabilities,^83,90^ it seems sensible to include this impact in the definition of severity.

A limited number of the included studies reported on the mechanism of the injury, namely a sudden or gradual onset. Due to the low number of studies for most sports and impairment categories, it is impossible to draw conclusions based on these results. However, for football 5-a-side, there seems to be a trend toward more acute injuries (Table 1).^74,86,92^ For powerlifting and track and field, a trend is seen for gradual onset injuries.^5,63,75,91,92^ Of course, these results should be interpreted with caution.

Overall, studies targeting recreational athletes were limited, even though most athletes with a physical impairment participate in recreational sports.^69,70^ This limitation is also visible in the types of sports and impairment categories. Given the number of sports and impairment categories in para-sports, it is doubtful that the included studies have covered all types and categories. For instance, the absence of research in non-Paralympic sports, such as fitness, yoga, etc, is notable. More research is needed to map the extent of recreational para-sports-related health problems.

### Risk Factors for Health Problems in Para-Sports

Regarding injury and illness etiology, we found some associations between incidence rate and risk factors. When we consider all risk factors, we see inconsistent findings among the studies. Because of the variety in definitions and the lack of an unequivocal method of quantifying variables, the results of the studies could not be pooled. Furthermore, all studies based the associations on the total injury numbers and made no distinctions between various injury types and their respective risk factors.

The factor “impairment related” was investigated in 34 studies, of which 14 reported a higher injury rate when the physical impairment was more severe. In some studies, this greater impairment was clear: a lower functional level in a certain impairment.^13,76,85^ Other studies that compared various impairment types, the difference in impairment was less clear.^5,52,72^ Which specific impairment has greater limitation in function is debatable and possibly also varies among individual participants. Therefore, we should regard these findings with caution.

The risk factor “age” was investigated in 19 studies. Most included studies focused on elite athletes, who are generally younger, and most comparisons between age groups did not include all possible age groups (Table 5). Therefore, we cannot draw information on injury occurrence in older athletes from these studies. The association between age and illness occurrence was investigated in 2 studies, both of high quality. There seems to be a consensus that older persons have a higher risk of illness than younger athletes (Table 5). However, we should interpret these results with caution due to the limited number of studies. It is also unclear whether this higher risk is influenced by sports participation or the normal effect of advancing age.

Some of the risk factors for sports injuries in nondisabled athletes that have been identified in the literature have thus far not been studied in para-sports, or only to a limited extent.^58^ The risk factor “history of previous injury,” which only 1 study investigated, is potentially very interesting because it is known from nondisabled sports that this factor is a great predictor of injury.^1,26-28,37,41^ Furthermore, equipment was investigated in a few studies, but this was limited to protective equipment (wheelchair cushions). None investigated the sports equipment, such as the adjustment of wheelchairs. Finally, environmental influences such as weather and snow or ice conditions or floor type have limited-to-no evidence.

### Prevention of Health Problems in Para-Sports

We identified only 3 studies with a high risk of bias on preventive measures, and all 3 focused on preventing shoulder complications in wheelchair athletes. These studies give a first insight into the potential of strengthening and stretching exercises of the shoulder muscles. Further research is needed to investigate these presumptions.

Although shoulder pain/injuries are frequent in wheelchair sports,^77^ preventive measures regarding other injuries have not been investigated. Furthermore, even though wheelchair sports are common, 80% of the reviewed studies included athletes participating in sports in a wheelchair. There is a gap in knowledge on preventive strategies for nonwheelchair athletes. Further research should focus on developing targeted preventive strategies for all the different impairment categories and types of sports.

### Strengths and Limitations

Selection bias may have occurred because only 1 author performed the title screening. Predetermined inclusion and exclusion criteria were used to limit this bias and, when in doubt, the article was brought to the abstract screening. Since 2 authors performed the abstract and full-text screening, we consider the risk of bias low. The exclusion of 11 non-English articles may have led to publication bias and missing useful information.

Because most participants in the included studies were of an elite level (81%), results may be limited to elite para-athletes. This has resulted in smaller sample sizes and varying results among studies. Furthermore, the included studies have a short follow-up because most research was performed during tournaments. Results obtained from elite athletes during tournaments cannot be generalized to athletes who perform year-round in elite or recreational sports.

## Conclusion

The current research addressing sports-related injuries and illnesses in para-sports shows a vast range in prevalence and incidence per sport and impairment category. Furthermore, there is limited evidence concerning the severity of these sports-related health problems. No consensus is yet reached in the literature when considering risk factors for sport-related health problems. The evidence regarding the development and effectiveness of preventive measures for para-athletes is sparse. The interpretation of the results is limited mainly to elite-level athletes during competitions, since there is little evidence concerning athletes of recreational level.

## Supplemental Material

sj-docx-1-sph-10.1177_19417381231178534 – Supplemental material for Sports-Related Health Problems in Para-Sports: A Systematic Review With Quality Assessment Supplemental material, sj-docx-1-sph-10.1177_19417381231178534 for Sports-Related Health Problems in Para-Sports: A Systematic Review With Quality Assessment by Sietske C.M. Luijten, Leonie M. te Loo, Joske Nauta, Thomas W.J. Janssen, Jasmijn F.M. Holla, René H.J. Otten, Ingrid Vriend and Evert Verhagen in Sports Health

sj-docx-2-sph-10.1177_19417381231178534 – Supplemental material for Sports-Related Health Problems in Para-Sports: A Systematic Review With Quality Assessment Supplemental material, sj-docx-2-sph-10.1177_19417381231178534 for Sports-Related Health Problems in Para-Sports: A Systematic Review With Quality Assessment by Sietske C.M. Luijten, Leonie M. te Loo, Joske Nauta, Thomas W.J. Janssen, Jasmijn F.M. Holla, René H.J. Otten, Ingrid Vriend and Evert Verhagen in Sports Health

sj-docx-3-sph-10.1177_19417381231178534 – Supplemental material for Sports-Related Health Problems in Para-Sports: A Systematic Review With Quality Assessment Supplemental material, sj-docx-3-sph-10.1177_19417381231178534 for Sports-Related Health Problems in Para-Sports: A Systematic Review With Quality Assessment by Sietske C.M. Luijten, Leonie M. te Loo, Joske Nauta, Thomas W.J. Janssen, Jasmijn F.M. Holla, René H.J. Otten, Ingrid Vriend and Evert Verhagen in Sports Health

sj-docx-4-sph-10.1177_19417381231178534 – Supplemental material for Sports-Related Health Problems in Para-Sports: A Systematic Review With Quality Assessment Supplemental material, sj-docx-4-sph-10.1177_19417381231178534 for Sports-Related Health Problems in Para-Sports: A Systematic Review With Quality Assessment by Sietske C.M. Luijten, Leonie M. te Loo, Joske Nauta, Thomas W.J. Janssen, Jasmijn F.M. Holla, René H.J. Otten, Ingrid Vriend and Evert Verhagen in Sports Health

sj-docx-5-sph-10.1177_19417381231178534 – Supplemental material for Sports-Related Health Problems in Para-Sports: A Systematic Review With Quality Assessment Supplemental material, sj-docx-5-sph-10.1177_19417381231178534 for Sports-Related Health Problems in Para-Sports: A Systematic Review With Quality Assessment by Sietske C.M. Luijten, Leonie M. te Loo, Joske Nauta, Thomas W.J. Janssen, Jasmijn F.M. Holla, René H.J. Otten, Ingrid Vriend and Evert Verhagen in Sports Health

sj-docx-6-sph-10.1177_19417381231178534 – Supplemental material for Sports-Related Health Problems in Para-Sports: A Systematic Review With Quality Assessment Supplemental material, sj-docx-6-sph-10.1177_19417381231178534 for Sports-Related Health Problems in Para-Sports: A Systematic Review With Quality Assessment by Sietske C.M. Luijten, Leonie M. te Loo, Joske Nauta, Thomas W.J. Janssen, Jasmijn F.M. Holla, René H.J. Otten, Ingrid Vriend and Evert Verhagen in Sports Health
