# Supplementary material for: Sports-Related Health Problems in Para-Sports: A Systematic Review With Quality Assessment
Source: Sports Health. 2023 Jun 19;16(4):551–64. doi: 10.1177/19417381231178534 (PMC11195855; doi:10.1177/19417381231178534)
Supplement: sj-docx-1-sph-10.1177_19417381231178534 – Supplemental material for Sports-Related Health Problems in Para-Sports: A Systematic Review With Quality Assessment [file sj-docx-1-sph-10.1177_19417381231178534.docx]

Appendix 1: Search strategy

**Search strategy in PubMed (2021 December 8^th^)**

| **#** | **Query** | **Results** |
| --- | --- | --- |
| **#3** | Search #1 AND #2 | **1168** |
| **#2** | Search "Disability Studies"[Mesh] OR "Disabled Persons"[Mesh:NoExp] OR "Amputees"[Mesh] OR "Disabled Children"[Mesh:NoExp] OR "Persons With Hearing Impairments"[Mesh] OR "Visually Impaired Persons"[Mesh] OR "Sports for Persons with Disabilities"[Mesh] OR Disability Stud*[tiab] OR Disabled Person*[tiab] OR Persons with Disabilit*[tiab] OR People with Disabilit*[tiab] OR Physically Handicapped[tiab] OR Physically Disabled[tiab] OR Physically Challenged[tiab] OR Amputee[tiab] OR Children with Disabil*[tiab] OR Handicapped Children[tiab] OR Disabled Child[tiab] OR Hearing Impaired Person*[tiab] OR Hearing Disabled Person*[tiab] OR Deaf Person*[tiab] OR Visually Impaired Person*[tiab] OR Blind Person*[tiab] OR Sports for the Disabled[tiab] OR Adaptive Sport*[tiab] OR Parathletic*[tiab] OR Para-athletic*[tiab] OR wheel-chair*[tiab] OR wheelchair*[tiab] OR paralympic*[tiab] OR adapted sport*[tiab] |  |
| **#1** | Search "Athletic Injuries"[Mesh] OR "Sports/injuries"[Mesh] OR "Exercise/injuries"[Mesh] OR ((athlet*[tiab] OR sport*[tiab] OR exercis*[tiab] OR physical activ*[tiab] OR motor activ*[tiab] OR locomotor activ*[tiab]) AND (injury[tiab] OR injuries[tiab] OR "injuries"[Subheading])) |  |

**Search strategy in Embase.com (2021 December 8^th^)**

| **#** | **Query** | **Results** |
| --- | --- | --- |
| **#3** | #1 AND #2 | **1415** |
| **#2** | 'disability study'/exp OR 'disabled person'/de OR 'handicapped child'/exp OR 'physically disabled person'/exp OR 'disabled sport'/exp OR 'disability stud*':ti,ab,kw OR 'disabled person*':ti,ab,kw OR 'persons with disabilit*':ti,ab,kw OR 'people with disabilit*':ti,ab,kw OR 'physically handicapped':ti,ab,kw OR 'physically disabled':ti,ab,kw OR 'physically challenged':ti,ab,kw OR 'amputee':ti,ab,kw OR 'children with disabil*':ti,ab,kw OR 'handicapped children':ti,ab,kw OR 'disabled child':ti,ab,kw OR 'hearing impaired person*':ti,ab,kw OR 'hearing disabled person*':ti,ab,kw OR 'deaf person*':ti,ab,kw OR 'visually impaired person*':ti,ab,kw OR 'blind person*':ti,ab,kw OR 'sports for the disabled':ti,ab,kw OR 'adaptive sport*':ti,ab,kw OR 'parathletic*':ti,ab,kw OR 'para-athletic*':ti,ab,kw OR 'wheel-chair*':ti,ab,kw OR 'wheelchair*':ti,ab,kw OR 'paralympic*':ti,ab,kw OR 'adapted sport*':ti,ab,kw |  |
| **#1** | 'sport injury'/exp OR ((athlet*:ti,ab,kw OR sport*:ti,ab,kw OR exercis*:ti,ab,kw OR 'physical activ*':ti,ab,kw OR 'motor activ*':ti,ab,kw OR 'locomotor activ*':ti,ab,kw) AND (injury:ti,ab,kw OR injuries:ti,ab,kw)) |  |

**Search strategy in SPORTDiscus (via EBSCO ; 2021 December 8^th^)**

| # | **Query** | **Results** |
| --- | --- | --- |
| S4 | S3  Limiters - Peer Reviewed | **924** |
| S3 | S1 AND S2 |  |
| S2 | DE "PEOPLE with disabilities" OR DE "AMPUTEES" OR DE "WOMEN amputees" OR DE "ATHLETES with disabilities" OR DE "AUTISTIC athletes" OR DE "BASEBALL players with disabilities" OR DE "BASKETBALL players with disabilities" OR DE "BLIND athletes" OR DE "DEAF athletes" OR DE "SKIERS with disabilities" OR DE "WOMEN athletes with disabilities" OR DE "CHILDREN with disabilities" OR DE "DEAFBLIND children" OR DE "DANCERS with disabilities" OR DE "WHEELCHAIR dancers" OR DE "DEAFBLIND people" OR DE "DEAFBLIND children" OR DE "DEVELOPMENTAL disabilities" OR DE "CEREBRAL palsy" OR DE "EPILEPSY" OR DE "TRAUMATIC epilepsy" OR DE "HEARING impaired" OR DE "DEAF" OR DE "MEN with disabilities" OR DE "PARALYTICS" OR DE "HEMIPLEGICS" OR DE "PARAPLEGICS" OR DE "PEOPLE with cerebral palsy" OR DE "QUADRIPLEGICS" OR DE "PEOPLE with visual disabilities" OR DE "BLIND" OR DE "BLIND athletes" OR DE "STUDENTS with disabilities" OR DE "WOMEN with disabilities" OR DE "WOMEN amputees" OR (DE "EXERCISE for people with disabilities" OR DE "AEROBIC exercises for people with visual disabilities" OR DE "GYMNASTICS for people with disabilities") OR (DE "SPORTS for people with disabilities" OR DE "ARCHERY for people with disabilities" OR DE "BASKETBALL for people with disabilities" OR DE "BOATING for people with disabilities" OR DE "CANOEING for people with disabilities" OR DE "KAYAKING for people with disabilities" OR DE "CRICKET for people with disabilities" OR DE "CYCLING for people with disabilities" OR DE "DIVING for people with disabilities" OR DE "DOGSLEDDING for people with disabilities" OR DE "FISHING for people with disabilities" OR DE "FOOTBALL for people with disabilities" OR DE "GOLF for people with disabilities" OR DE "GYMNASTICS for people with disabilities" OR DE "HANDBALL for people with disabilities" OR DE "HANG gliding for people with disabilities" OR DE "HOCKEY for people with disabilities" OR DE "HUNTING for people with disabilities" OR DE "MARTIAL arts for people with disabilities" OR DE "MOTORCYCLING for people with disabilities" OR DE "MOTORSOCCER" OR DE "PARACHUTING for people with disabilities" OR DE "ROCK climbing for people with disabilities" OR DE "RUGBALL" OR DE "RUNNING for people with disabilities" OR DE "SHOOTING for people with disabilities" OR DE "SKATING for people with disabilities" OR DE "SKIING for people with disabilities" OR DE "SKYDIVING for people with disabilities" OR DE "SNOWSHOEING for people with disabilities" OR DE "SOCCER for people with disabilities" OR DE "SOFTBALL for people with disabilities" OR DE "SPORTS for children with disabilities" OR DE "SPORTS for people with visual disabilities" OR DE "GOALBALL" OR DE "SURFING for people with disabilities" OR DE "SWIMMING for people with disabilities" OR DE "TENNIS for people with disabilities" OR DE "VOLLEYBALL for people with disabilities" OR DE "SITTING volleyball" OR DE "WEIGHT training for people with disabilities" OR DE "WHEELCHAIR sports" OR DE "BOCCIA (Game)" OR DE "MOTORSOCCER" OR DE "NATIONAL Veterans Wheelchair Games" OR DE "RUGBALL" OR DE "WHEELCHAIR basketball" OR DE "WHEELCHAIR basketball -- Competitions" OR DE "WHEELCHAIR bowling" OR DE "WHEELCHAIR dance sport" OR DE "WHEELCHAIR fencing" OR DE "WHEELCHAIR hockey" OR DE "WHEELCHAIR road racing" OR DE "WHEELCHAIR rugby" OR DE "WHEELCHAIR soccer" OR DE "WHEELCHAIR sports competitions" OR DE "WHEELCHAIR tennis" OR DE "WHEELCHAIR track & field" OR DE "WHEELCHAIR workouts" OR DE "WINDSURFING for people with disabilities" OR DE "WORLD Games for the Deaf") OR TI (“Disability Stud*” OR “Disabled Person*” OR “Persons with Disabilit*” OR “People with Disabilit*” OR “Physically Handicapped” OR “Physically Disabled” OR “Physically Challenged” OR “Amputee” OR “Children with Disabil*” OR “Handicapped Children” OR “Disabled Child” OR “Hearing Impaired Person*” OR “Hearing Disabled Person*” OR “Deaf Person*” OR “Visually Impaired Person*” OR “Blind Person*” OR “Sports for the Disabled” OR “Adaptive Sport*” OR “Parathletic*” OR “Para-athletic*” OR “wheel-chair*” OR “wheelchair*” OR “paralympic*” OR “adapted sport*”) OR AB (“Disability Stud*” OR “Disabled Person*” OR “Persons with Disabilit*” OR “People with Disabilit*” OR “Physically Handicapped” OR “Physically Disabled” OR “Physically Challenged” OR “Amputee” OR “Children with Disabil*” OR “Handicapped Children” OR “Disabled Child” OR “Hearing Impaired Person*” OR “Hearing Disabled Person*” OR “Deaf Person*” OR “Visually Impaired Person*” OR “Blind Person*” OR “Sports for the Disabled” OR “Adaptive Sport*” OR “Parathletic*” OR “Para-athletic*” OR “wheel-chair*” OR “wheelchair*” OR “paralympic*” OR “adapted sport*”) OR SU (“Disability Stud*” OR “Disabled Person*” OR “Persons with Disabilit*” OR “People with Disabilit*” OR “Physically Handicapped” OR “Physically Disabled” OR “Physically Challenged” OR “Amputee” OR “Children with Disabil*” OR “Handicapped Children” OR “Disabled Child” OR “Hearing Impaired Person*” OR “Hearing Disabled Person*” OR “Deaf Person*” OR “Visually Impaired Person*” OR “Blind Person*” OR “Sports for the Disabled” OR “Adaptive Sport*” OR “Parathletic*” OR “Para-athletic*” OR “wheel-chair*” OR “wheelchair*” OR “paralympic*” OR “adapted sport*”) |  |
| S1 | DE "SPORTS injuries" OR DE "ACHILLES tendinitis" OR DE "AEROBICS injuries" OR DE "AQUATIC sports injuries" OR DE "BOATING injuries" OR DE "YACHTING injuries" or DE "DIVING injuries" OR DE "SCUBA diving injuries" OR DE "SKIN diving injuries" OR DE "SWIMMING injuries" OR DE "YACHTING injuries" OR DE "BASEBALL injuries" OR DE "SOFTBALL injuries" OR DE "BASKETBALL injuries" OR DE "BOXING injuries" OR DE "COMMOTIO cordis" OR DE "CRICKET injuries" OR DE "DELAYED onset muscle soreness" OR DE "EQUESTRIAN accidents" OR DE "FOOTBALL injuries" OR DE "GOLF injuries" or DE "GOLF elbow" OR DE "GYMNASTICS injuries" OR DE "HIKING injuries" OR DE "HOCKEY injuries" or DE "FIELD hockey injuries" OR DE "HORSE sports injuries" OR DE "IN-line skating injuries" OR DE "JOGGING injuries" OR DE "JUDO injuries" OR DE "JUMPER'S knee" OR DE "KARATE injuries" OR DE "MARTIAL arts injuries" or DE "HAND-to-hand fighting injuries" OR DE "MOTORSPORTS injuries" OR DE "NETBALL injuries" OR DE "RACKET game injuries" OR DE "SQUASH injuries" OR DE "RUGBY football injuries" OR DE "RUNNING injuries" OR DE "MORTON'S foot" OR DE "SHIN splints" OR DE "SKATEBOARDING injuries" OR DE "SOCCER injuries" OR DE "TENNIS injuries" OR DE "TENNIS elbow" OR DE "TURF toe" OR DE "VAULTING injuries" OR DE "VOLLEYBALL injuries" OR DE "WALKING (Sports) injuries" OR DE "WEIGHT training injuries" OR DE "WINTER sports injuries" OR DE "SKIING injuries" OR DE "CROSS-country skiing injuries" OR DE "SNOWBOARDING injuries" OR TI ((athlet* OR sport* OR exercis* OR “physical activ*” OR “motor activ*” OR “locomotor activ*”) AND (injury OR injuries)) OR AB ((athlet* OR sport* OR exercis* OR “physical activ*” OR “motor activ*” OR “locomotor activ*”) AND (injury OR injuries)) OR SU ((athlet* OR sport* OR exercis* OR “physical activ*” OR “motor activ*” OR “locomotor activ*”) AND (injury OR injuries)) |  |

**Search strategy in CINAHL (via EBSCO ; 2021 December 8^th^)**

| # | **Query** | **Results** |
| --- | --- | --- |
| S3 | S1 AND S2 | **1078** |
| S2 | (MH "Disabled") OR (MH "Amputees") OR (MH "Athletes, Disabled") OR (MH "Child, Disabled") OR (MH "Students, Disabled") OR (MH "Parents, Disabled") OR TI (“Disability Stud*” OR “Disabled Person*” OR “Persons with Disabilit*” OR “People with Disabilit*” OR “Physically Handicapped” OR “Physically Disabled” OR “Physically Challenged” OR “Amputee” OR “Children with Disabil*” OR “Handicapped Children” OR “Disabled Child” OR “Hearing Impaired Person*” OR “Hearing Disabled Person*” OR “Deaf Person*” OR “Visually Impaired Person*” OR “Blind Person*” OR “Sports for the Disabled” OR “Adaptive Sport*” OR “Parathletic*” OR “Para-athletic*” OR “wheel-chair*” OR “wheelchair*” OR “paralympic*” OR “adapted sport*”) OR AB (“Disability Stud*” OR “Disabled Person*” OR “Persons with Disabilit*” OR “People with Disabilit*” OR “Physically Handicapped” OR “Physically Disabled” OR “Physically Challenged” OR “Amputee” OR “Children with Disabil*” OR “Handicapped Children” OR “Disabled Child” OR “Hearing Impaired Person*” OR “Hearing Disabled Person*” OR “Deaf Person*” OR “Visually Impaired Person*” OR “Blind Person*” OR “Sports for the Disabled” OR “Adaptive Sport*” OR “Parathletic*” OR “Para-athletic*” OR “wheel-chair*” OR “wheelchair*” OR “paralympic*” OR “adapted sport*”) OR SU (“Disability Stud*” OR “Disabled Person*” OR “Persons with Disabilit*” OR “People with Disabilit*” OR “Physically Handicapped” OR “Physically Disabled” OR “Physically Challenged” OR “Amputee” OR “Children with Disabil*” OR “Handicapped Children” OR “Disabled Child” OR “Hearing Impaired Person*” OR “Hearing Disabled Person*” OR “Deaf Person*” OR “Visually Impaired Person*” OR “Blind Person*” OR “Sports for the Disabled” OR “Adaptive Sport*” OR “Parathletic*” OR “Para-athletic*” OR “wheel-chair*” OR “wheelchair*” OR “paralympic*” OR “adapted sport*”) |  |
| S1 | (MH "Athletic Injuries+") OR TI ((athlet* OR sport* OR exercis* OR “physical activ*” OR “motor activ*” OR “locomotor activ*”) AND (injury OR injuries)) OR AB ((athlet* OR sport* OR exercis* OR “physical activ*” OR “motor activ*” OR “locomotor activ*”) AND (injury OR injuries)) OR SU ((athlet* OR sport* OR exercis* OR “physical activ*” OR “motor activ*” OR “locomotor activ*”) AND (injury OR injuries)) |  |

**Search strategy in the Cochrane Library (via Wiley ; 2021 December 8^th^)**

| **#** | **Query** | **Results** |
| --- | --- | --- |
| **#6** | #3 and (#4 or #5) | **149** |
| **#5** | embase:an |  |
| **#4** | pubmed:an |  |
| **#3** | #1 and #2 |  |
| **#2** | “disability stud*”:ti,ab,kw OR “disabled person*”:ti,ab,kw OR “persons with disabilit*”:ti,ab,kw OR “people with disabilit*”:ti,ab,kw OR “physically handicapped”:ti,ab,kw OR “physically disabled”:ti,ab,kw OR “physically challenged”:ti,ab,kw OR “amputee”:ti,ab,kw OR “children with disabil*”:ti,ab,kw OR “handicapped children”:ti,ab,kw OR “disabled child”:ti,ab,kw OR “hearing impaired person*”:ti,ab,kw OR “hearing disabled person*”:ti,ab,kw OR “deaf person*”:ti,ab,kw OR “visually impaired person*”:ti,ab,kw OR “blind person*”:ti,ab,kw OR “sports for the disabled”:ti,ab,kw OR “adaptive sport*”:ti,ab,kw OR “parathletic*”:ti,ab,kw OR “para-athletic*”:ti,ab,kw OR “wheel-chair*”:ti,ab,kw OR “wheelchair*”:ti,ab,kw OR “paralympic*”:ti,ab,kw OR “adapted sport*”:ti,ab,kw |  |
| **#1** | ((athlet*:ti,ab,kw OR sport*:ti,ab,kw OR exercis*:ti,ab,kw OR "physical activ*":ti,ab,kw OR "motor activ*":ti,ab,kw OR "locomotor activ*":ti,ab,kw) AND (injury:ti,ab,kw OR injuries:ti,ab,kw)) |  |
