# Supplementary material for: Sports-Related Health Problems in Para-Sports: A Systematic Review With Quality Assessment
Source: Sports Health. 2023 Jun 19;16(4):551–64. doi: 10.1177/19417381231178534 (PMC11195855; doi:10.1177/19417381231178534)
Supplement: sj-docx-2-sph-10.1177_19417381231178534 – Supplemental material for Sports-Related Health Problems in Para-Sports: A Systematic Review With Quality Assessment [file sj-docx-2-sph-10.1177_19417381231178534.docx]

*Appendix 2: Included studies consisting injury information according to step 1 of the Sequence of Prevention*

| **STUDY** | **TITLE** | | **STUDY DESIGN** | **INJURY DEFINITION** | **SPORT** | **FOLLOW-UP DURATION** | **SAMPLE SIZE** | **DISABILITY TYPE** | **PREVALENCE** | **INCIDENCE** | **SEVERITY** | **SUDDEN** | **GRADUAL** |
| --- | --- | --- | --- | --- | --- | --- | --- | --- | --- | --- | --- | --- | --- |
| **BURNHAM ET AL. 1991 [9]** | | Sports Medicine for the Physically Disabled: The Canadian Team Experience at the 1988 Seoul Paralympic Games | 1 | Medical attention | Paralympic summer sports | 10 days | 151 | 1, 2 | 82% |  |  |  |  |
| **BURNHAM ET AL. 1993 [10]** | | Shoulder pain in wheelchair athletes - The role of muscle imbalance | 2 | A shoulder was defined as having rotator cuff impingement syndrome if it was painful to the athlete and had at least two of the clinical signs on physical examination | Basketball, road racing and weight training | N/A | 19 Wheelchair athletes and 20 able-bodied athletes | 1 | 26% |  |  |  |  |
| **CALMELS ET AL. 1994 [12]** | | Medical activity during an international sporting competition for the physically disables: Saint-Etienne World Handicapped Sport Championships | 1 | Medical consultations | Paralympic summer sports | 11 days | 1200 | 1, 2, 3 | 6.6% |  |  |  |  |
| **REYNOLDS ET AL. 1994 [70]** | | Paralympics - Barcelona 1992 | 1 | Medical attention | Paralympic summer sports | 2 weeks | 205 | 1, 2, 3 | 69% |  |  |  |  |
| **FERRARA & BUCKLEY. 1996 [33]** | | Athletes With Disabilities Injury Registry | 1 | An injury was when a scheduled practice or competition was modified, missed, or interrupted due to an injury, illness, or pain for 1 day or more | Paralympic summer sports | 2 years | 319 | 1, 2, 3 |  | 9.45 per 1000 athlete exposures | 52% injuries were minor (time-loss of 7 days or less), 29% moderate (8-21 days) and 19% major (22 or more days) |  |  |
| **MIYAHARA & GERRARD. 1998 [62]** | | The Relationship of Strength and Muscle Balance to Shoulder Pain and Impingement Syndrome in Elite Quadriplegic Wheelchair Rugby Players | 2 | Shoulder pain | Wheelchair rugby | N/A | 8 | 1 | 25% |  |  |  |  |
| **FERRARA ET AL. 2000 [34]** | | A longitudinal study of injuries to athletes with disabilities | 1 | Any injury/illness that was evaluated by the US medical staff during these competitions | Paralympic sports | 60 days (total of all competition days) | 220 (WC), 345 (PT), 360 (PGI), 55 (AC), 380 (PGII) | 1, 2, 3 | 5.0% (WC), 16.4% (PT), 37.3% (PGI), 2.1% (AC)39.2% (PGII) |  |  | 67.9% | 20.6% |
| **NYLAND ET AL. 2000 [64]** | | Soft Tissue Injuries to USA Paralympians at the 1996 Summer Games | 1 | Soft tissue injuries were operationally defined as strain, sprain, tendonitis, bursitis, or contusion | Paralympic summer sports | 1996 Summer Paralympic Games | 304 | 1, 2, 3 |  |  |  | 67% | 33% |
| **WEBBORN ET AL. 2006 [90]** | | Injuries among Disabled Athletes during the 2002 Winter Paralympic Games | 1 | NR | Paralympic winter sports | 20 days | 416 | 1, 2 | 9% |  | 27% of acute injuries were time loss injuries | 77% | 15% |
| **SILVA ET AL. 2011 [78]** | | Aspects of Sports injuries in Athletes with Visual Impairment | 1 | Any injury which has occurred with the athlete during practice, training or competition which causes interruption, limitation or alteration in his/her participation for one or more days | Track and Field, Soccer 5, Goalball, Judo and Swimming | 4 years | 131 | 2 |  | 2.82 injuries per athlete over the 4 year period |  | 47.22% | 52.78% |
| **CHUNG ET AL. 2012 [13]** | | Musculoskeletal Injuries in Elite Able-Bodied and Wheelchair Foil Fencers - A Pilot Study | 1 | Trauma that occurred during a training/competition and prohibited the athlete from continuing fencing activity for at least 1 day | Wheelchair foil fence | 3 years | 14 | 1 |  | 3.9 (3.1 - 4.7) per 1000 hours of exposure | Among the 7 cat B fencers, 4 were absent from training/competition for more than 22 days due to partial-thickness tendon tear of their fencing shoulders | 61.1% | 38.9% |
| **SILVA ET AL. 2012 [75]** | | Sports Injuries in Brazilian Blind Footballers | 1 | Any injury that caused an athlete to stop, limit, or modify participation for one or more days | 5-a-side football | 4 years | 13 | 2 |  | 0.12 injuries per match |  | 80% | 20% |
| **WEBBORN ET AL. 2012 [89]** | | The Injury Experience at the 2010 Winter Paralympic Games | 3 | Any sports-related musculoskeletal complaint that caused the athlete to seek medical attention during the study period, regardless of the athlete's ability to continue with training or competition | Paralympic winter sports | Duration of 2010 Winter Paralympic Games | 505 | 1, 2 |  | IP 23.8% (20.1 - 27.7) |  | 40.8% | 57.5% |
| **DERMAN ET AL. 2013 [22]** | | Illness and injury in athletes during the competition period at the London 2012 Paralympic Games: development and implementation of a web-based surveillance system (WEB-IISS) for team medical staff | 1 | Any newly acquired injury as well as exacerbations of pre-existing injury that occurred during training and/or competition of the 14 day pre-competition and competition period of the London 2012 Paralympic Games | Paralympic summer sports | 14 days | 3565 | NR | 11.6% | 12.7 (11.7 - 13.7)* |  | 52% | 30% |
| **WILLICK ET AL. 2013 [93]** | | The epidemiology of injuries at the London 2012 Paralympic Games | 1 | Any sport-related musculoskeletal or neurological complaint prompting an athlete to seek medical attention, regardless of whether or not the complaint resulted in lost time from training or competition | Paralympic summer sports | 14 days | 3565 | 1, 2 |  | 12.7 (11.7 - 13.7)* |  | 51.5% | 31.8& |
| **SILVA ET AL. 2013 [77]** | | Sports Injuries in Paralympic Track and Field Athletes with Visual Impairment | 3 | Any injury that caused an athlete to stop, limit, or modify participation for 1 day or more | Track and Field | 4 years | 40 | 2 | 78% | 1.93 per participating athlete |  | 18% | 82% |
| **SILVA ET AL. 2013 [76]** | | Sport Injuries in Elite Paralympic Swimmers With Visual Impairment | 3 | Any injury that caused an athlete to stop, limit, or modify participation for one or more days | Swimming | 4 years | 28 | 2 | 64% | 1.5 injuries per participating athlete and 0.3 injuries per match |  | 20% | 80% |
| **GAWRÓNKSI ET AL. 2013 [40]** | | Fit and healthy Paralympians - medical care guidelines for disabled athletes: a study of the injuries and illnesses incurred by the Polish Paralympic team in Beijing 2008 and London 2012 | 1 | A newly acquired musculoskeletal symptom or an exacerbation of a pre-existing (chronic) injury that occurred during training and/or competition | Paralympic summer sports | 21 days in Beijing and 16 days in London | 91 (Beijing) 100 (London) | 1, 2, 3 |  | 29.8 (22.1 - 37.6)* (B), 15 (9 – 21)* (L) |  | 12.6 (7.5 - 17.6)* (B), 8.8 (4.2 - 13.3)* (L) | 17.3 (11.4 - 23.2)* (B), 6.3 (2.4 – 21)* (L) |
| **BAUERFEIND ET AL. 2015 [4]** | | Sports injuries in wheelchair rugby - a pilot study | 1 | NR | Wheelchair rugby | 9 months | 14 | 1 |  | 0,3 per player per training day |  |  |  |
| **WILLICK ET AL. 2015 [92]** | | The epidemiology of injuries in powerlifting at the London 2012 Paralympic Games: An analysis of 1411 athlete-days | 1 | Any newly acquired injury as well as exacerbations of pre-existing injury that occurred during training and/or competition of the 14 day pre-competition and competition period of the London 2012 Paralympic Games | Powerlifting | 7 days | 163 | 1 |  | 33.3* |  | 13.2% | 60.5% |
| **WEBBORN ET AL. 2016 [87]** | | The Epidemiology of Injuries in Football at the London 2012 Paralympic Games | 1 | Any newly acquired injury as well as exacerbations of pre-existing injury that occurred during training and/or competition of the 14 day pre-competition and competition period of the London 2012 Paralympic Games | Football | 14 days | 166 | 1, 2 |  | 5-a-side: 22.4 (14.1 - 33.8)*  7-a-side 10.4 (5.4 - 15.5)* | 7-a-side 14 injuries (50%) resulted in less than 1 day of time loss. 5 injuries resulted in more than 1 day lost from training or competition | 5-a-side: 54,5% 7-a-side: 71.4% | 5-a-side: 22.7% 7-a-side: 7.1% |
| **DERMAN ET AL. 2016 [24]** | | High incidence of injury at the Sochi 2014 Winter Paralympic Games: a prospective cohort study of 6564 athlete days | 1 | Any newly acquired injury as well as exacerbations of pre-existing injury that occurred during training and/or competition of the Games period of the Sochi 2014 Winter Paralympic Games | Paralympic winter sports | 12 days | 547 | 1, 2 | 24.5% | 26.5 (22.7 - 30.8)* | 20.1% resulted in time loss of 1 or more days | 67% | 18% |
| **BLAUWET ET AL. 2016 [5]** | | Risk of Injuries in Paralympic Track and Field Differs by Impairment and Event Discipline | 1 | Any newly acquired injury as well as exacerbations of pre-existing injury that occurred during training and/or competition of the 14 day pre-competition and competition period of the London 2012 Paralympic Games | Track and Field | 10 days | 977 | 1, 2, 3 |  | 22.1 (19.5 - 24.7)* | Ambulant athletes: 6.7 (4.8-8.6)* Wheelchair/seated athletes: 4.1 (1.9-6.2)* | 50.3% (Ambulant), 49.1% (WC) | 28.9% (Ambulant), 29.8% (WC) |
| **FAGHER ET AL. 2017 [32]** | | An eHealth application of self-reported sports-related injuries and illnesses in Paralympic sport: pilot feasibility and usability study | 1, 5 | Any new musculoskeletal pain, feeling, or injury that causes changes in normal training or competition to the mode, duration, intensity, or frequency, regardless of whether or not time is lost from training or competition | Shooting, canoeing, goalball, athletics, judo, swimming, boccia, cycling, table tennis, wheelchair rugby, cross-country skiing, wheelchair curling, ice hockey | 4 weeks | 21 | 1, 2, 3 |  | 1.8 per 100 hours | The typical injury severity was 1 to 3 days of time loss of training and 2.6 missed training sessions for illnesses. | 20% | 80% |
| **KASINSKA & TASIEMSKI. 2017 [53]** | | Determinants of sports injuries in amputee football: initial analysis | 1 | NR | Football | 6 months | 40 | 1 |  | 0.045 per player per training day |  |  |  |
| **DERMAN ET AL. 2017 [20]** | | High precompetition injury rate dominates the injury profile at the Rio 2016 Summer Paralympic Games: a prospective cohort study of 51198 athlete days | 1 | Any newly acquired injury as well as exacerbations of pre-existing injury that occurred during training and/or competition of the Games period of the Rio 2016 Summer Paralympic Games | Paralympic summer sports | 14 days | 3657 | 1, 2, 3 |  | 10 (9.1 - 10.9)* | 128 injuries (25.1%) resulted in time loss. 90 injuries resulted in time loss of 2 or more days. In total 7.7 days lost per 1000 athlete days | 51.8% | 34.5% |
| **KUBOSCH ET AL. 2017 [55]** | | Upcoming Paralympic Summer Games in Rio - what did the German medical team learn from the London Games? | 1 | Any musculoskeletal complaint or exacerbation of pre-existing musculoskeletal complaints that occurred during training and/or competition during the 14-day pre-competition and competition period of the London 2012 Paralympic Games | Paralympic summer sports | 22 days | 150 | 1, 2 | 93.3% | 64.3 (± 15.4)* | 43 cases (20%) the ability to perform was limited and 4 athletes (3%) were unable to continue | 7% | 93% |
| **HOLLANDER ET AL. 2019 [48]** | | Epidemiology of injuries during the Wheelchair Basketball World Championships 2018: A prospective cohort study | 1 | Any newly incurred musculo-skeletal complaint (traumatic or overuse) and/or concussion during the tournament receiving medical attention regardless of the consequences for participation | Wheelchair basketball | 11 days | 132 | 1 |  | 68.9 (55.4 - 82.4)* | 8 time loss injuries. 6.1 (1.9-10.3) injuries per 100 players. | 40% | 52% |
| **ONA AYALA ET AL. 2019 [65]** | | Injury epidemiology and preparedness in powerlifting at the Rio 2016 Paralympic Games: An analysis of 1410 athlete-days | 1 | Any newly acquired injury as well as exacerbations of pre-existing injury that occurred during training and/or competition of the 3-day pre-competition and 7-day competition period at the Rio 2016 Paralympic Games | Paralympic summer sports | 10 days | 141 | 1 |  | 15.6 (9.61 - 21.59)* |  | 18% | 64% |
| **PÉREZ-TEJERO & GÓMEZ. 2019 [68]** | | Shoulder pain assessment in elite wheelchair basketball players | 2 | Shoulder pain | Wheelchair basketball | 5 days | 17 | 1 | 52.9% had SP according to the SPI-WB but only 35.3% had SP according to the clinical test |  |  |  |  |
| **DERMAN ET AL. 2020 [19]** | | High incidence of injuries at the Pyeongchang 2018 Paralympic Winter Games: a prospective cohort study of 6804 athlete days | 1 | Any newly acquired injury as well exacerbations of pre-existing injury that occurred during training and/or competition of the Games period of the Pyeongchang 2018 Paralympic Winter Games | Paralympic winter sports | 12 days | 567 | 1, 2 | 19.8% | 20.9 (17.4 - 25.0)* | 21.1% of injuries resulted in time loss. 3 injuries were moderately serious (8-28 days lost) and 3 were serious (28 days-6 months lost) | 77% | 15% |
| **CYR ET AL. 2020 [17]** | | Prevalence of lateral epicondylosis in manual wheelchair users participating in adaptive sports | 2 | The primary outcome measure was the prevalence of CET in manual wheelchair users based on ultrasound  assessment or LE based on physical exam. Participants met diagnostic criteria for CET via ultrasound assessment by meeting two of three diagnostic criteria: (1) tendon thickening, (2) increased vascularity, and (3) hypo echogenicity | Wheelchair sports | N/A | 87 | 1 | 46% met LE criteria via ultrasound. 17% met LE criteria for physical exam |  |  |  |  |
| **FAGHER ET AL. 2020 [30]** | | Injuries and illnesses in Swedish Paralympic athletes-A 52-week prospective study of incidence and risk factors | 1 | “Any new musculoskeletal pain, feeling, injury, illness, or psychological complaint that caused changes in normal training or competition to the mode, duration, intensity, or frequency, regardless of whether or not time was lost from training or competition”. | Summer and Winter Paralympic sports | 52 weeks | 107 | 1, 2, 3 | Annual incidence proportion: 68% | 6.9 injuries per 1000 hours of sports exposure | The time loss from sport due to injury (severity) was as follows: 0-3 days (33%), 4-7 days (24%), 8-20 days (10%), ≥21 days (23%), and ≥ 3 months (11%) | 32% | 68% |
| **MEIRELLES ET AL. 2020 [61]** | | The prevalence of carpal tunnel syndrome in adapted Sports athletes based on clinical diagnostic. | 2 | They hypothesized for this study that the presence of two or more signs and/or symptoms characterizes the clinical diagnosis of carpal tunnel syndrome. | Weightlifting, wheelchair fencing, seated volleyball, wheelchair basketball, capoeira, wheelchair table tennis | N/A | 72 | 1 | 8% |  |  |  |  |
| **TAMAI ET AL. 2020 [81]** | | The relationship between skin ultrasound images and muscle damage using skin blotting in wheelchair basketball athletes | 2 | Skin structural features were classified into four categories, namely, normal skin structure (three images), unclear superficial and deep fascia (nine images), cloudy fat layer (six images), and fat infiltration and low-echoic lesion/anechoic lesion (six images) | Basketball | N/A | 12 | 1 | 5/12 has a presence of deep tissue damage after imaging. 2/12 after visual inspection. |  |  |  |  |
| **BUSCH ET AL. 2021 [11]** | | Health Problems in German Paralympic Athletes Preparing for the 2020 Tokyo Paralympic Games. | 1 | Any condition that reduces an athlete’s normal state of full health. Disorders of the musculoskeletal system and concussions were classified as injuries and further subcategorized into acute (onset linked to a specific injury event) or overuse injuries (no specific injury event | Summer Paralympic sports | 10 months | 79 | 1, 2 |  | 3.4 injuries per 1000 hours of exposure | Total health problems: time loss in days (mean) 6.6 (13.7). Acute 13 (23.8) overuse 4.2 (7), illness 4.6 (6.4), multiple health problem 3.6 (3.3) | 1.8 (1.3-2.3) acute injuries per 1000 hours of exposure | 1.6 (1.2 - 2.2) overuse injuries per 1000 hours of exposure |
| **BRANCALEONE ET AL. 2021 [6]** | | Concussion Epidemiology in Athletes Who Are Deaf or Hard-of-Hearing Compared With Athletes Who Are Hearing | 3 | An athlete was considered to have a concussion if a “postinjury 1” ImPACT assessment was completed. | Varsity football, soccer, basketball, baseball, and softball | 5 years | 693 athletes who are deaf or hard of hearing and 1284 athletes who are hearing | 5 | 4.33% (2.78 - 5.88) |  |  |  |  |
| **HIRSCHMÜLLER ET AL. 2021 [46]** | | Injury and Illness Surveillance in Elite Para Athletes: An Urgent Need for Suitable Illness Prevention Strategies | 1 | Disorders of the musculoskeletal system as well as concussions were classified as injuries and further subcategorized into acute (onset linked to a specific injury event) and overuse injuries (no specific injury event) | Summer Paralympic sports | 29 weeks | 58 | 1, 2, 3 | Weekly prevalence: 18% | 7.6 injuries per 1000 hours of exposure | 149 (31%) days lost due to overuse and 31 (6%) due to acute injuries | 2.8 (2.0-4.0) acute injuries per 1000 athlete days | 4.8 (3.6-6.2) overuse injuries per 1000 athlete days |
| **KASITINON ET AL. 2021 [54]** | | Health-Related Incidents among Intercollegiate Wheelchair Basketball Players | 1 | Self-reported injury | Wheelchair basketball | 5.5. months | 28 | 1, 4 |  | 12.7 (9.1- 16.3) per 1000 exposure hours | 33% of injuries resulted in time loss | 31.3% | 39.6% |
| **LEXELL ET AL. 2021 [56]** | | Incidence of sports-related concussion in elite para athletes–a 52-week prospective study | 1 | an SRC was recorded: (i) if the athlete had a blow to or jolting of the head that had caused an acute disruption of brain function with concomitant symptoms; (ii) if it had caused changes in normal training or competition regarding the mode, duration, intensity, or frequency; and (iii) if it was confirmed by medical personnel. | Summer and Winter Paralympic sports | 52 weeks | 107 | 1, 2, 3 | Incidence proportion: 9.3% (4.8-16.7%) | 0.5 SRC/1000 hours of sport exposure (0.3-0.9) | Five of the SRC (38%) lead to a time loss from participation in normal training and sport for more than 21 days. Four (31%) lead to a time loss for 8–20 days, and the remaining four SRC (n = 4; 31%) lead to a time loss for 4–7 days. |  |  |
| **MAURICE ET AL. 2021 [59]** | | The 2019 Cameroon University Games: Prevention Strategies for Musculoskeletal Injuries | 1 | "Musculoskeletal injury was defined as any physical symptom that required medical attention, or prevented an athlete  from taking full part in training and/or competition. These included all injuries that received medical attention, or caused performance restriction or time loss to the athletes’  training/competition and were newly incurred during the Games." | Para-athletics, power-lifting and para-tennis | 2019 Cameroon University Games | 97 |  | 0 | 0 |  |  |  |
| **SANTOS ET AL. 2021 [73]** | | 5-A-Side Soccer: Prevalence Of Sports Injuries In Brazilian Team Players | 3 | "Sports injury (SI): tissue injury or other disturbance of normal physical  function due to participation in sports, resulting from repetitive or rapid  transfer of kinetic energy" | 5-a-side soccer | N/A | 8 | 2 | 62.5% | 0.87 injuries per athletes per year | 4 injuries led to more than 21 days lost. 1 injury led to 2 days lost and 2 injuries to training adaptations but no lost days. | 43% | 57% |
| **STEFFEN ET AL. 2021 [80]** | | Illness and injury among Norwegian Para athletes over five consecutive Paralympic Summer and Winter Games cycles: prevailing high illness burden on the road from 2012 to 2020 | 1 | Any reported health problem, irrespective of its consequences on their sports participation or performance, and irrespective of whether they had sought medical attention | Summer and Winter Paralympic sports | 5 Paralympic cycles (winter and summer) | 94 | 1, 2, 3 | Weekly avg prevalence injuries: 19.8 [18.9-20.7] | 2.7 injuries per athlete per year | 5 days lost due to acute injuries (range 0-121 days), 9 days due to overuse injuries (0-145) | 34% | 66% |
| **YAMAGUCHI ET AL. 2021 [96]** | Injuries and Illnesses Observed in Athletes from Beginner to Elite Levels at the 18th National Sports Festival for Persons with Disabilities in Japan | | 3 | Medical attention injuries | Archery, Athletics, Basketball, Bowling, Flying disc, Foot baseball, Grand softball, Soccer, Softball, Swimming, Table tennis, Volleyball, Wheelchair basketball | 7 days | 3277 athletes participate in the Festival (information on 136 athletes due to visits medical stations) | 1, 2, 3, 4, 5 |  | 11.8* |  |  |  |
| **GUTIÉRREZ-SANTIAGO ET AL. 2020 [41]** | Sport Injuries in Elite Paralympic Judokas: Findings From the 2018 World Championship | | 1 | “any new musculoskeletal pain, feeling or injury derived from competition circumstances that caused alteration and/or interruption of normal combat whether in the mode, duration, intensity, or frequency, regardless of whether or not time is lost from competition.” | Judo | IBSA 2018 World Judo Championship | 267 | 2 | Incidence proportion: 18.9 (14.8-24.2) injuries per 100 fighters | 68.5 (52.5-89.2) per 1000 athlete exposures | Incidence rate severe injuries: 14.8 (8.2-26.5) per 1000 athlete exposures |  |  |
| NOTE: Study design; 1) prospective cohort design 2) cross-sectional study 3) descriptive study 4) observational study 5) feasibility study 6) clinical trial/RCT. Disability type; 1) physically disabled 2) Visually disabled 3) Intellectual disabled 4) Mental health condition 5) Hearing impairment. *IR per 1000 athlete days | | | | | | | | | | | | |  |
