# Supplementary material for: Sports-Related Health Problems in Para-Sports: A Systematic Review With Quality Assessment
Source: Sports Health. 2023 Jun 19;16(4):551–64. doi: 10.1177/19417381231178534 (PMC11195855; doi:10.1177/19417381231178534)
Supplement: sj-docx-3-sph-10.1177_19417381231178534 – Supplemental material for Sports-Related Health Problems in Para-Sports: A Systematic Review With Quality Assessment [file sj-docx-3-sph-10.1177_19417381231178534.docx]

*Appendix 3: Included studies consisting illness information according to step 1 of the Sequence of Prevention*

| **STUDY** | **TITLE** | **STUDY DESIGN** | **ILLNESS DEFINITION** | **SPORT** | **FOLLOW-UP DURATION** | **SAMPLE SIZE** | **DISABILITY TYPE** | **PREVALENCE** | **INCIDENCE** | **SEVERITY** |
| --- | --- | --- | --- | --- | --- | --- | --- | --- | --- | --- |
| **CALMELS ET AL. 1994 [12]** | Medical activity during an international sporting competition for the physically disables: Saint-Etienne World Handicapped Sport Championships | 1 | NR | Para summer sports | 11 days | 1200 | 1, 2, 3 | 5.2% |  |  |
| **DERMAN ET AL. 2013 [22]** | Illness and injury in athletes during the competition period at the London 2012 Paralympic Games: development and implementation of a web-based surveillance system (WEB-IISS) for team medical staff | 1 | Any newly acquired illness as well as exacerbations of pre-existing illness that occurred during training and/or competition or during or immediately before the 2012 Paralympic Games | London 2012 Paralympic Games | 14 days | 3565 | NR | 10.2 % | 13.2 (12.2 - 14.2)* |  |
| **GAWRONKSI ET AL. 2013 [40]** | Fit and healthy Paralympians - medical care guidelines for disabled athletes: a study of the injuries and illnesses incurred by the Polish Paralympic team in Beijing 2008 and London 2012 | 1 | Any newly-acquired non-injury (or acute exacerbation of a pre-existing or chronic complaint) | Paralympic summer sports | 21 days in Beijing and 16 days in London | 91 (Beijing) 100 (London) | 1, 2, 3 |  | 49.2 (39.2 - 59.1)* (B), 31.3 (22.6 - 39.9)* (L) |  |
| **DERMAN ET AL. 2014 [21]** | Clinical Characteristics of 385 Illnesses of Athletes With Impairment Reported on the WEB-IISS System During the London 2012 Paralympic Games | 1 | Any newly required illness as well as exacerbations of pre-existing illness that occurred during training or competition, and during or immediately before the London 2012 Paralympic Games | Paralympic summer sports | 14 days | 3329 | 1, 2, 3 | 9.2% | 8.3 (7.5 - 9.1)* | 9.9% illnesses resulted in 1 day lost, 9.6% illnesses resulted in more than 1 day of time loss |
| **DERMAN ET AL. 2016 [25]** | The incidence and patterns of illness at the Sochi 2014 Winter Paralympic Games: a prospective cohort study of 6564 athlete days | 1 | Any newly acquired illness as well as exacerbations of pre-existing illness that occurred during training and/or competition or during or immediately before the Sochi 2014 Winter Paralympic Games | Paralympic winter sports | 12 days | 547 | 1, 2, 3 |  | 18,7 (15,1 - 23,2)* | 21% of the athletes reported an illness that required 1 or more days of time loss |
| **FAGHER ET AL. 2017 [32]** | An eHealth application of self-reported sports-related injuries and illnesses in Paralympic sport: pilot feasibility and usability study | 1, 5 | Any new illness or psychological complaint that causes changes in normal training or competition to the mode, duration, intensity, or frequency, regardless of whether or not time is lost from training or competition | Shooting, canoeing, goalball, athletics, judo, swimming, boccia, cycling, table tennis, wheelchair rugby, cross-country skiing, wheelchair curling, ice hockey | 4 weeks | 21 | 1, 2, 3 |  | 1.7 per 100 hours |  |
| **DERMAN ET AL. 2018 [23]** | Sport, sex and age increase risk of illness at the Rio 2016 Summer Paralympic Games: a prospective cohort study of 51198 athlete days | 1 | Any athlete requiring medical attention for an illness regardless of the consequences with regard to absences from training or competition | Paralympic summer sports | 14 days | 3657 | 1, 2, 3 |  | 10.0 (9.2 - 10.9)* | 84 illnesses resulted in time-loss (16.4%) for 1 day or more |
| **FAGHER ET AL. 2020 [30]** | Injuries and illnesses in Swedish Paralympic athletes-A 52-week prospective study of incidence and risk factors | 1 | “Any new musculoskeletal pain, feeling, injury, illness, or psychological complaint that caused changes in normal training or competition to the mode, duration, intensity, or frequency, regardless of whether or not time was lost from training or competition”. | Summer and Winter Paralympic sports | 52 weeks | 107 | 1, 2, 3 | Annual incidence proportion: 77% | 9.3 illnesses per 1000 hours of sports exposure | The severity (time loss) of illnesses were as follows: 0-3 days (38%), 4-7 days (42%), 8-20 days (14%), ≥21 days (5%), and ≥ 3 months (2%). |
| **BUSCH ET AL. 2021 [11]** | Health Problems in German Paralympic Athletes Preparing for the 2020 Tokyo Paralympic Games. | 1 | Any condition that reduces an athlete’s normal state of full health. Disorders of other body systems were classified as illnesses. | Summer Paralympic sports | 10 months | 79 | 1, 2 |  | 0.6 (0.5-0.7 per 100 athlete days | Total health problems: time loss in days (mean) 6.6 (13.7). Acute 13 (23.8) overuse 4.2 (7), illness 4.6 (6.4), multiple health problem 3.6 (3.3) |
| **HIRSCHMÜLLER ET AL. 2021 [46]** | Injury and Illness Surveillance in Elite Para Athletes: An Urgent Need for Suitable Illness Prevention Strategies | 1 | Disorders of the musculoskeletal system as well as concussions were classified as injuries and further subcategorized into acute (onset linked to a specific injury event) and overuse injuries (no specific injury event), disorders of other body system were classified as illnesses | Summer Paralympic sports | 29 weeks | 58 | 1, 2, 3 | Weekly prevalence: 13% (11%-15%) | 8.6 (7.0-10.5) illnesses per 1000 athlete days | 306 days lost (63%) due to illnesses |
| **STEFFEN ET AL. 2021 [80]** | Illness and injury among Norwegian Para athletes over five consecutive Paralympic Summer and Winter Games cycles: prevailing high illness burden on the road from 2012 to 2020 | 1 | Any reported health problem, irrespective of its consequences on their sports participation or performance, and irrespective of whether they had sought medical attention | Summer and Winter Paralympic sports | 5 Paralympic cycles (winter and summer) | 94 | 1, 2, 3 | Weekly average prevalence illnesses: 19.1 [18.0-20.3] | 4.8 (4.4-5.3) injuries per athlete per year | On average 18 days lost due to illness (0-61) |
| NOTE: Study design; 1) prospective cohort design 2) cross-sectional study 3) descriptive study 4) observational study 5) feasibility study 6) clinical trial/RCT. Disability type; 1) physically disabled 2) Visually disabled 3) Intellectual disabled 4) Mental health condition 5) Hearing impairment. *IR per 1000 athlete days. | | | | | | | | | | |
