# Supplementary material for: Sports-Related Health Problems in Para-Sports: A Systematic Review With Quality Assessment
Source: Sports Health. 2023 Jun 19;16(4):551–64. doi: 10.1177/19417381231178534 (PMC11195855; doi:10.1177/19417381231178534)
Supplement: sj-docx-4-sph-10.1177_19417381231178534 – Supplemental material for Sports-Related Health Problems in Para-Sports: A Systematic Review With Quality Assessment [file sj-docx-4-sph-10.1177_19417381231178534.docx]

*Appendix 4: Included studies consisting aetiology information according to step 2 of the Sequence of Prevention*

| **STUDY** | **TITLE** | **INJURY/illness DEFiNITION** | **SPORT** | **FOLLOW-UP DURATION** | **SAMPLE SIZE** | **DISABILITY TYPE** | **risk factors** | **SIGNIFICANT ASSOCIATIONS** | **NON-SIGNIFICANT ASSOCIATIONS** | **QUALITY ASSESSMENT** |
| --- | --- | --- | --- | --- | --- | --- | --- | --- | --- | --- |
| **Burnham et al. 1991 [9]** | Sports Medicine for the Physically Disabled: The Canadian Team Experience at the 1988 Seoul Paralympic Games | Medical attention | Paralympic summer sports | 10 days | 151 | 1, 2 | Impairment related | More lower extremity injuries in blind athletes | High number of back injuries in athletes with cerebral palsy | 4 |
| **Burnham et al. 1993 [10]** | Shoulder pain in wheelchair athletes - The role of muscle imbalance | A shoulder was defined as having rotator cuff impingement syndrome if it was painful to the athlete and had at least two of the clinical signs on physical examination | Basketball, road racing and weight training | NR | 19 Wheelchair athletes and 20 able-bodied athletes | 1 | Muscle strength  Impairment related  Limb dominance | Paraplegic athletes were stronger in shoulder abduction, adduction, and external and internal rotation compared to able-bodied athletes. Ratio shoulder abduction to adduction strength was sing higher in paraplegic compared to able-bodied  Shoulder of paraplegics with impingement syndrome were sign weaker in adduction and internal and external rotation and had a relative weakness of the internal rotators compared to no impingement | No difference in strength balance  No difference between dominant and non-dominant side | 3 |
| **Ferrara & Buckley. 1996 [33]** | Athletes With Disabilities Injury Registry | An injury was when a scheduled practice or competition was modified, missed, or interrupted due to an injury, illness, or pain for 1 day or more | Paralympic summer sports | 2 years | 319 | 1, 2, 3 | Coping |  | There was a greater percentage of time-loss injuries for athletes who were coached than those who were self-coached | 7 |
| **Miyahara & Gerrard. 1998 [62]** | The Relationship of Strength and Muscle Balance to Shoulder Pain and Impingement Syndrome in Elite Quadriplegic Wheelchair Rugby Players | Shoulder pain | Wheelchair rugby | 2 weeks | 8 | 1 | Muscle strength  Range of motion  Impairment related  Age  Training load | The measure of external rotation at 180 degrees predicted 75% of shoulder pain with external rotation)  Functional class predicted 81,25% of shoulder with tenderness over the lesser tuberosity) (the combi of functional classification and the hours spent in sports activity predicted 87,5% of shoulders with tenderness over the greater tuberosity  Age predicted 75% of the painful shoulders with external rotation, 68,75% of shoulder with tenderness over the acromioclavicular joint, and 75% of the shoulders with tenderness over the biceps tendon  The hours spent in sports activity predicted 87,5% of shoulder with tenderness over anterior acromion and 75,5% of shoulders with wasting of the supra- or infraspinatus fossae | The strength and demographic variables could not distinguish the shoulders diagnosed with impingement syndrome from the rest of the shoulders | 5 |
| **CURTIS & BLACK, 1999 [16]** | Shoulder Pain in Female Wheelchair Basketball Players | Shoulder pain | Wheelchair basketball | NR | 46 | 1 | Wheelchair use | Athletes driving more hours per week had higher WUSPI scores.  Athletes who performed more transfers a day had higher WUSPI scores | Ambulatory athletes report higher mean WUSPI score compared to full-time wheelchair users | 2 |
| **Nyland et al. 2000 [64]** | Soft Tissue Injuries to USA Paralympians at the 1996 Summer Games | Soft tissue injuries were operationally defined as strain, sprain, tendonitis, bursitis, or contusion | Paralympic summer sports | 1996 Summer Paralympic Games | 304 | 1, 2, 3 | Impairment related |  | The different sports associations who facilitate sports for various disability categories show differences in injury location | 8 |
| **fullerton, Borckardt & alfano, 2003 [37]** | Shoulder Pain: A Comparison of Wheelchair Athletes and Nonathletic Wheelchair Users | Shoulder pain | Wheelchair sports | NR | 257 | 1 | Impairment related  Age  Sports experience | The older subjects reported more shoulder pain  Athletes had lower shoulder pain rates compared to non-athletes. Non-athletes developed shoulder pain 4 years earlier compared to athletes | No relation between pain status, athlete status and years in a wheelchair with shoulder pain. No difference between paraplegic and quadriplegic athletes and shoulder pain  No relation between number of years athletes had been involved in wheelchair sports and pain status | 3 |
| **finley & Rodgers, 2004 [36]** | Prevalence and identification of shoulder pathology in athletic and nonathletic wheelchair users with shoulder pain: A pilot study | Shoulder pain | NR | NR | 52 | 1, 2 | Muscle strength  Range of motion |  | No significant relation between shoulder pain and strength  No significant relation between shoulder pain and range of motion | 3 |
| **Webborn et al. 2006 [90]** | Injuries among Disabled Athletes during the 2002 Winter Paralympic Games | NR | Paralympic winter sports | 20 days | 416 | 1, 2 | Sex  Equipment  Type of sports |  | The overall and sport-specific injury rates did not differ sign by gender  7 out of 39 cases felt the cause of injury was equipment, but this was not statistically tested  All Nordic skiing injuries involved the upper limb. For alpine skiing 33% of the injuries involved the upper limb and 38%the lower limb | 6 |
| **Jeon et al, 2010 [52]** | Ultrasonographic Evaluation of the Shoulder in Elite Wheelchair Tennis Players | Shoulder pain | Wheelchair tennis | NR | 33 | 1 | Age  Sports experience |  | No differences in age between acromioclavicular and supraspinatus pathology  No differences in length of wheelchair use between acromioclavicular and supraspinatus pathology.  No differences in training time per day and length of career as a wheelchair tennis player between acromioclavicular and supraspinatus pathology | 4 |
| **Silva et al. 2011 [78]** | Aspects of Sports injuries in Athletes with Visual Impairment | Any injury which has occurred with the athlete during practice, training or competition which causes interruption, limitation or alteration in his/her participation for one or more days | Track and Field, Soccer 5, Goalball, Judo and Swimming | 4 years | 131 | 2 | Impairment related  Sex | Sign difference between injury rates between visual level B1 and B3 | Female athletes suffered from more injuries than male athletes, however, this difference was not significant | 7 |
| **Chung et al. 2012 [13]** | Musculoskeletal Injuries in Elite Able-Bodied and Wheelchair Foil Fencers - A Pilot Study | Trauma that occurred during a training/competition and prohibited the athlete from continuing fencing activity for at least 1 day | Wheelchair foil fence | 3 years | 14 | 1 | Impairment related  Competition phase | Wheelchair fencers had higher rates compared to able bodied fencers (higher risk of minor injury, muscle strain and shoulder and elbow injury), and the wheelchair fencers without active trunk control had higher rates compared to active trunk control group  Higher injury rates during competition compared to training |  | 9 |
| **Webborn et al. 2012 [89]** | The Injury Experience at the 2010 Winter Paralympic Games | Any sports-related musculoskeletal complaint that caused the athlete to seek medical attention during the study period, regardless of the athlete's ability to continue with training or competition | Paralympic winter sports | Duration of 2010 Winter Paralympic Games | 505 | 1, 2 | Type of sports  Sex |  | Higher incidences in sledge hockey and alpine skiing but there was no significant relation  No significant difference in injury incidence proportion between men and women | 9 |
| **Derman et al. 2013 [22]** | Illness and injury in athletes during the competition period at the London 2012 Paralympic Games: development and implementation of a web-based surveillance system (WEB-IISS) for team medical staff | Any newly acquired injury as well as exacerbations of pre-existing injury that occurred during training and/or competition of the 14 day pre-competition and competition period of the London 2012 Paralympic Games | Paralympic summer sports | 14 days | 3565 | NR | Competition phase |  | No significant difference in injury and illness rates during pre-competition compared to competition | 8 |
| **Willick et al. 2013 [93]** | The epidemiology of injuries at the London 2012 Paralympic Games | Any sport-related musculoskeletal or neurological complaint prompting an athlete to seek medical attention, regardless of whether or not the complaint resulted in lost time from training or competition | Paralympic summer sports | 14 days | 3565 | 1, 2 | Sex  Age  Type of sports  Competition phase | For female athletes pre-competition injury rates were significantly higher compared to competition injury rates | No difference in the injury incidence in female compared to male athletes  The highest rates in the 26-34 age group, but significance was not reached  No differences in the injury rates between different sports | 9 |
| **Silva et al. 2013 [77]** | Sports Injuries in Paralympic Track and Field Athletes with Visual Impairment | Any injury that caused an athlete to stop, limit, or modify participation for 1 day or more | Track and Field | 4 years | 40 | 2 | Impairment related  Sex |  | Differences in prevalence between visual classifications but not sign, T/F11 highest, followed by T/F12 and T/F13  Females had higher injury rates but not significant | 8 |
| **Silva et al. 2013 [76]** | Sport Injuries in Elite Paralympic Swimmers With Visual Impairment | Any injury that caused an athlete to stop, limit, or modify participation for one or more days | Swimming | 4 years | 28 | 2 | Impairment related    Sex |  | Higher prevalence rates in S13, followed by S11 and S12 and higher clinical incidence in S11 followed by S13 and S12  Injury prevalence was slightly higher in females but not significantly. Clinical incidence was slightly higher in males compared to females but also not significantly | 8 |
| **Gawronski, Sobiecka & Malesza, 2013 [40]** | Fit and healthy Paralympians - medical care guidelines for disabled athletes: a study of the injuries and illnesses incurred by the Polish Paralympic team in Beijing 2008 and London 2012 | A newly acquired musculoskeletal symptom or an exacerbation of a pre-existing (chronic) injury that occurred during training and/or competition | Paralympic summer sports | 21 days in Beijing and 16 days in London | 91 (Beijing) 100 (London) | 1, 2, 3 | Impairment related |  | Athletes witch spinal cord injury had the highest injury rates, but not significantly different from other disability categories | 9 |
| **Derman et al. 2014 [21]** | Clinical Characteristics of 385 Illnesses of Athletes With Impairment Reported on the WEB-IISS System During the London 2012 Paralympic Games | Any newly required illness as well as exacerbations of pre-existing illness that occurred during training or competition, and during or immediately before the London 2012 Paralympic Games | Paralympic summer sports | 14 days | 3329 | 1, 2, 3 | Impairment related |  | The highest  number of all illnesses, were of athletes with spinal cord injury. | 9 |
| **mutsuzaki et al. 2014 [63]** | Factors associated with deep tissue injury in male wheelchair basketball players of a Japanese national team | Deep tissue injury is defined as injury to soft tissue resulting from pressure and/or shear | Wheelchair basketball | NR | 20 | 1 | Body weight  Impairment related  Sports experience  Wheelchair use | Low-echoic lesions rates were higher in athletes with spinal cord injury compared to skeletal system disease  Players who used a wheelchair in daily life had higher rates of low-echoic lesions compared to players who use it only for sports | No significant association between low-echoic lesions and BMI  No significant association between low-echoic lesions and length of athletic career.  No sign association between low-echoic lesions and basketball class | 6 |
| **Bauerfeind et al. 2015 [4]** | Sports injuries in wheelchair rugby - a pilot study | NR | Wheelchair rugby | 9 months | 14 | 1 | Aggressiveness  Player position |  | Offensive players had higher levels of aggressiveness compared to defensive players furthermore, no significant association was found between level of anger and aggressiveness and injury incidence  Offensive players experiences more injuries that were not consulted by a physician compared to defensive players | 9 |
| **Willick et al. 2015 [92]** | The epidemiology of injuries in powerlifting at the London 2012 Paralympic Games: An analysis of 1411 athlete-days | Any newly acquired injury as well as exacerbations of pre-existing injury that occurred during training and/or competition of the 14 day pre-competition and competition period of the London 2012 Paralympic Games | Powerlifting | 7 days | 163 | 1 | Body weight  Sex  Age | Lighter weight class (IP 34.6%) had significantly more injuries compared to heavier athletes (IP 17.3%) | No significant difference in jury rates between male and female powerlifters  Highest rates were found in the age group 26-34 followed by the youngest group (13-25) and the oldest (35-67) | 9 |
| **Webborn et al. 2015 [87]** | The Epidemiology of Injuries in Football at the London 2012 Paralympic Games | Any newly acquired injury as well as exacerbations of pre-existing injury that occurred during training and/or competition of the 14 day pre-competition and competition period of the London 2012 Paralympic Games | Football | 14 days | 166 | 1, 2 | Age  Competition phase  Player position |  | There was a trend towards a higher injury rate in 26-34 years olds compared to younger and older athletes  Majority of injuries occurred during competition, significance was not reached  Goalkeeper had IR 0 injuries (0-18.6) and outfielders had IR 28.1 (17.7-42.2), but difference not significantly determined in football 5-a-side | 9 |
| **aytar et al. 2015 [2]** | Scapular resting position, shoulder pain and function in disabled athletes | NR | Wheelchair basketball, amputee soccer, disabled table tennis | NR | 63 | 1 | Wheelchair use  Type of sports | Significant differences in shoulder pain between sports | Wheelchair using sports athletes have higher % of abnormal scapular resting position, whereas crutch -using sports athletes have better scapular resting position  No differences for shoulder pain, function and dyskinesia between wheelchair basketball and disables table tennis players.  Pain reported is slightly higher in wheelchair basketball.  Amputee soccer players have better perceived shoulder functionality compared to wheelchair using sports athletes | 3 |
| **Derman et al. 2016 [25]** | The incidence and patterns of illness at the Sochi 2014 Winter Paralympic Games: a prospective cohort study of 6564 athlete days | Any newly acquired illness as well as exacerbations of pre-existing illness that occurred during training and/or competition or during or immediately before the Sochi 2014 Winter Paralympic Games | Paralympic winter sports | 12 days | 547 | 1, 2, 3 | Sex  Age  Type of sports | Older subjects had significant highest illness rates compared to younger athletes | No significant difference in illness incidence proportion between men and women  Highest total illnesses is in alpine skiing but highest IR is in wheelchair curling | 9 |
| **Derman et al. 2016 [24]** | High incidence of injury at the Sochi 2014 Winter Paralympic Games: a prospective cohort study of 6564 athlete days | Any newly acquired injury as well as exacerbations of pre-existing injury that occurred during training and/or competition of the Games period of the Sochi 2014 Winter Paralympic Games | Paralympic winter sports | 12 days | 547 | 1, 2 | Sex  Age  Type of sports | The older age groups had significant higher rates  Alpine skiing/snowboarding had significant higher rates compared to other sports, namely cross-country skiing/biathlon, ice sledge hockey, wheelchair curling | No significant difference in injury incidence proportion between men and women | 9 |
| **Blauwet et al. 2016 [5]** | Risk of Injuries in Paralympic Track and Field Differs by Impairment and Event Discipline | Any newly acquired injury as well as exacerbations of pre-existing injury that occurred during training and/or competition of the 14 day pre-competition and competition period of the London 2012 Paralympic Games | Track and Field | 10 days | 977 | 1, 2, 3 | Impairment related  Sex  Age  Type of sports  Competition phase | Significant difference between athletes with visual impairments and amputation compared to CP in track disciplines. In field ambulant throwing categories there were significant higher rates in short stature compared to other impairment groups.  Significant higher acute rates compared to chronic in ambulant track and field athletes  Male athletes had sign higher rates compared to females in track disciplines  In field categories, there were significant higher rates in seated throwing compared to wheelchair racing.  Higher non-time-loss injuries compared to time-loss injuries in ambulant and seated track and field athletes  Significant higher rates during competition compared to training in both ambulant and seated athletes | Ambulant athletes had no significant differences between track and field disciplines.  No differences between the acute and chronic injury rates for seated athletes  In field disciplines no differences between males and females  No differences between age groups | 9 |
| **Tsunoda et al., 2016 [82]** | Correlates of shoulder pain in wheelchair basketball players from the Japanese national team: A cross-sectional study | Shoulder pain | Wheelchair basketball | NR | 40 | 1 | Impairment related  Sex  Age  Sports experience | Lower ability was in men significantly correlated to greater shoulder pain  Higher WUSPI rates in men compared to women  In men, higher age was significantly correlated with greater shoulder pain  In women, longer practice time was correlated with less shoulder pain.  In men there was a significant correlation between more years of experience and shoulder pain |  | 2 |
| **Fagher et al. 2017 [32]** | An eHealth application of self-reported sports-related injuries and illnesses in paralympic sport: pilot feasibility and usability study | Any new musculoskeletal pain, feeling, or injury that causes changes in normal training or competition to the mode, duration, intensity, or frequency, regardless of whether or not time is lost from training or competition | Shooting, canoeing, goalball, athletics, judo, swimming, boccia, cycling, table tennis, wheelchair rugby, cross-country skiing, wheelchair curling, ice hockey | 4 weeks | 21 | 1, 2, 3 | Impairment related |  | No significant differences were reported between different impairment categories | 6 |
| **Shimizu et al., 2017 [74]** | A survey of deep tissue injury in elite female wheelchair basketball players | Deep tissue injury (DTI) | Wheelchair basketball | NR | 22 | 1 | Impairment related  Sports experience  Wheelchair use | DTI findings were more frequent in athletes with central nervous system disorder compared to skeletal.  DTI findings were frequent in sacral regions for CNS disorders compared to skeletal  The players with pelvic instability class 1.0 to 2.5 had sign more DTI findings compared to class 3.0 to 4.5  Lower systolic blood pressure, lower RBC counts and lower Cr levels are correlated with increased DTI findings  More players who used a wheelchair in daily life had DTIs compared to those who only use it for basketball | No differences in DTI findings in the ischium region  No association between DTI and length of athletic career | 7 |
| **Kasinska & tasiemski. 2017 [53]** | Determinants of sports injuries in amputee football: initial analysis | NR | Football | 6 months | 40 | 1 | Aggressiveness  Impairment related    Equipment  Player position  Sports experience | Athletes with congenital disability had higher rates compared to acquired disabilities  Defenders had significant lower rates compared to forwards and goalkeepers  The longer the training experience, the less injuries | No significant correlations of injury rates with competitive aggressiveness and anger  Most injuries were caused by crutches, but not significantly  No differences between forwards and goalkeepers | 8 |
| **Derman et al. 2018 [20]** | High pre-competition injury rate dominates the injury profile at the Rio 2016 Summer Paralympic Games: a prospective cohort study of 51198 athlete days | Any newly acquired injury as well as exacerbations of pre-existing injury that occurred during training and/or competition of the Games period of the Rio 2016 Summer Paralympic Games | Paralympic summer sports | 14 days | 3657 | 1, 2, 3 | Impairment related  Sex  Age  Type of sports  Competition phase | Significant higher time loss between the age group 26-34 and 35-75  Significant higher rates in football 5-a-side, judo and football 7-a-side, additionally, significant lower rates in boccia and para swimming  Significant higher rates during pre-competition compared to competition period | Highest rates in athletes with limb deficiency followed by visual impairment, spinal cord injury and central neurological impairment  No significant difference in the injury incidence in female compared to male athletes  No significant injury rate differences between age groups | 9 |
| **Kubosch et al. 2016 [55]** | Upcoming Paralympic Summer Games in Rio - what did the German medical team learn from the London Games? | Any musculoskeletal complaint or exacerbation of pre-existing musculoskeletal complaints that occurred during training and/or competition during the 14-day pre-competition and competition period of the London 2012 Paralympic Games | Paralympic summer sports | 22 days | 150 | 1, 2 | Impairment related  Sex  Age  Type of sports  Competition phase |  | No significant difference in disability category between injured and uninjured athletes  No significant difference in sex between injured and uninjured athletes  No significant difference in age between injured and uninjured athletes  No significant difference in injury rates between the various sports  Most injuries happened in training (87%) rather than competition (6%) or leisure time (7%), but no significance reported | 9 |
| **warner et al., 2018 [86]** | Scapular kinematics in professional wheelchair tennis players | NR | Wheelchair tennis | NR | 43 | 1 | Impairment related | Wheelchair players had sign more upwardly rotated scapula when comparing the dominant side than the able bodied impingement group and more upwardly rotated at 90 degrees of humeral elevation in the scapular plane. Also sign differences in scapular kinematics when comparing the non-dominant side. These differences in posterior tilt may reduce the risk of impingement in wheelchair players |  | 2 |
| **Derman et al. 2018 [23]** | Sport, sex and age increase risk of illness at the Rio 2016 Summer Paralympic Games: a prospective cohort study of 51198 athlete days | Any athlete requiring medical attention for an illness regardless of the consequences with regard to absences from training or competition | Paralympic summer sports | 14 days | 3657 | 1, 2, 3 | Sex  Age  Type of sports | Females had significant higher rates compared to males  The older age groups had significant higher rates and more time loss  Wheelchair fencing, para swimming, wheelchair basketball had significant higher rates compared to other sports |  | 9 |
| **Hollander et al. 2019 [48]** | Epidemiology of injuries during the Wheelchair Basketball World Championships 2018: A prospective cohort study | Any newly incurred musculo-skeletal complaint (traumatic or overuse) and/or concussion during the tournament receiving medical attention regardless of the consequences for participation | Wheelchair basketball | 11 days | 132 | 1 | Sex  Competition phase | Significant difference in mechanism between males and females, males had more contact injuries with other players and females had more non-contact injuries  Significant difference in injury mechanism between training and competition | No gender differences comparing proportion  No significant difference between training and competition regarding location, sex and type of injury | 8 |
| **Ona Ayala et al. 2019 [65]** | Injury epidemiology and preparedness in powerlifting at the Rio 2016 Paralympic Games: An analysis of 1410 athlete-days | Any newly acquired injury as well as exacerbations of preexisting injury that occurred during training and/or competition of the 3-day pre-competition and 7-day competition period at the Rio 2016 Paralympic Games | Paralympic summer sports | 10 days | 141 | 1 | Body weight  Sex  Age | A significant trend of increasing incidence rate of injuries as age increases | There was no significant difference in injury rate when comparing the heavier and lighter weight classes in men and women  No significant difference in injury rate between male and female powerlifters | 8 |
| **Pérez-tejero & Gómez. 2019 [68]** | Shoulder pain assessment in elite wheelchair basketball players | Shoulder pain | Wheelchair basketball | 5 days | 17 | 1 | Range of motion  Impairment related    Age | Athletes with less ROM reported more shoulder pain during ADLs and sport skills.  Significant negative correlation between shoulder pain according to the impingement test and ROM | Only the higher classes (4-4.5) have less shoulder pain  Shoulder pain related to ADL and sport more frequent in age group 20-30 years but not significantly | 2 |
| **Derman et al. 2020 [19]** | High incidence of injuries at the Pyeongchang 2018 Paralympic Winter Games: a prospective cohort study of 6804 athlete days | Any newly acquired injury as well exacerbations of pre-existing injury that occurred during training and/or competition of the Games period of the Pyeongchang 2018 Paralympic Winter Games | Paralympic winter sports | 12 days | 567 | 1, 2 | Impairment related  Sex  Age  Type of sports  Competition phase | Higher rates in para snowboard compared to the other sports, Wheelchair curling had significant lower rates compared to all other sports except Nordic skiing | 53.8% of the injuries were in athletes with limb deficiency and 26.9% in spinal cord injury athletes, but no significant difference reported  No significant difference in injury incidence proportion between men and women  No significant differences between age groups  No significant differences between pre-competition and competition | 9 |
| **CYR et al. 2020 [17]** | Prevalence of lateral epicondylosis in manual wheelchair users participating in adaptive sports | Participants met diagnostic criteria for CET via ultrasound assessment by meeting two of three diagnostic criteria: (1) tendon thickening, (2) increased vascularity, and (3) hypoechogenicity | Wheelchair sports | the 2018 and 2019 National Veteran Wheelchair Games | 87 | 1 | Age  Wheelchair use | Using logistic regression models, increased age was significant predictors for ultrasound findings suggestive of LE  Using logistic regression models number years of wheelchair use was significant predictors for ultrasound findings suggestive of LE |  | 6 |
| **Fagher et al. 2020 [30]** | Injuries and illnesses in Swedish Paralympic athletes-A 52-week prospective study of incidence and risk factors | “Any new musculoskeletal pain, feeling, injury, illness, or psychological complaint that caused changes in normal training or competition to the mode, duration, intensity, or frequency, regardless of whether or not time was lost from training or competition”. | Summer and Winter Paralympic sports | 52 weeks | 107 | 1, 2, 3 | Impairment related  Type of sports  Overtraining and stress  Sex  Previous injury  Training load | Wheelchair athletes and athletes with SCI reported more injuries to the upper extremities and ambulatory athletes more injury in the lower extremities. Athletes with VI reported more multiple injuries.  Log-rank tests showed statistically significant variations in SP with regard to type of sport  Log-rank tests showed statistically significant variations in SP with regard to gender  Log-rank tests showed statistically significant variations in SP with regard to previous severe injury  Athletes with a middle TLRI (Training load rand index) reported a significantly (P = .019) higher IR (22 illnesses/1000 hours) | The athletes reported that the cause of illness was due to overtraining and stress (45%) | 8 |
| **Heneghan et al. 2020 [43]** | Injury surveillance in elite Paralympic athletes with limb deficiency: a retrospective analysis of upper quadrant injuries | self-referred to a physiotherapist with an upper quadrant injury, defined as ‘tissue damage or other derangement of normal physical function due to participation in sports, resulting from rapid or repetitive transfer of kinetic energy’ | Powerlifting, Para-Archery, Wheelchair Basketball, Para-Cycling, Para-Canoe, Para-Triathlon, Para-Sailing, Para-Shooting, and Para-Swimming | 8 years | 34 | 1 | Impairment related  Range of motion  Posture  Age  Training load  Competition/training phase |  | No differences were seen between congenital and traumatic limb loss groups. Athletes with quadruple limb deficiency (n = 2) had double the number of injuries compared to those with single level of limb deficiency  In terms of physiotherapist findings on examination, joint stiffness was reported most frequently-  -Followed by posture  Athletes in the 20–29 age range experienced more injuries than other age groups with 4 injuries per athlete  Training volume or intensity was reported most frequently  More than half (?) the injuries occurred directly from training (58%, n = 94) or competition (9%, n = 15) | 5 |
| **Meirelles et al. 2020 [61]** | The prevalence of carpal tunnel syndrome in adapted Sports athletes based on clinical diagnostic. | They hypothesized for this study that the presence of two or more signs and/or symptoms characterizes the clinical diagnosis of carpal tunnel syndrome. | Weightlifting, wheelchair fencing, seated volleyball, wheelchair basketball, capoeira, wheelchair table tennis | NA | 72 | 1 | Wheelchair use  Type of sports  Limb dominance  Sports experience |  | No statistical relationship between the presence of symptoms and the use of wheelchairs  No statistical relationship between the presence of symptoms with the type of sport practiced  No statistical relationship between the presence of symptoms and dominance  No statistical relationship between the presence of symptoms and how long time the sport had been practiced. | 6 |
| **gutiérrez-santiago et al. 2020 [41]** | Sport Injuries in Elite Paralympic Judokas: Findings From the 2018 World Championship | “any new musculoskeletal pain, feeling or injury derived from competition circumstances that caused alteration and/or interruption of normal combat whether in the mode, duration, intensity, or frequency, regardless of whether or not time is lost from competition.” | Judo | IBSA 2018 World Judo Championship | 267 | 2 | Impairment related  Sex  Body mass |  | No significant differences were detected between the visual classes in any of the analyzed variables.  The relative risk analysis showed that sex and weight were  potential risk factors in the analyzed sample (non-significant) | 7 |
| **Busch et al. 2021 [11]** | Health Problems in German Paralympic Athletes Preparing for the 2020 Tokyo Paralympic Games. | "Health problems were defined following the International Olympic Committee consensus statement as any  condition that reduces an athlete’s normal state of full health. Disorders of the musculoskeletal system and concussions were classified as injuries“ | Summer Paralympic sports | 10 months | 79 | 1, 2 | Weather/season |  | A season effect with higher incidences of illnesses can be seen during the winter months in late 2019 and early 2020. | 8 |
| **Brancaleone et al. 2021 [6]** | Concussion Epidemiology in Athletes Who Are Deaf or Hard-of-Hearing Compared With Athletes Who Are Hearing | An athlete was considered to have a concussion if a “postinjury 1” ImPACT assessment was completed. | Varsity football, soccer, basketball, baseball, and softball | 5 years | 693 athletes Deaf/Hard of Hearing and 1284 athletes who are hearing | 5 | Impairment related  Type of sports | For all sports combined, athletes who are hearing had an significant increased concussion rate compared with athletes who are D/HoH (IRR 5 1.87, 95% CI, 1.26-2.78) | No difference in concussion rate was found between athletes who are D/HoH and athletes who are hearing when examining sex-comparable sports | 5 |
| **heneghan et al. 2021 [44]** | Lumbosacral injuries in elite Paralympic athletes with limb deficiency: a retrospective analysis of patient records | Medical attention injuries, lumbosacral injuries | Powerlifting, para-archery, wheelchair basketball, para-cycling, para-canoe, para-triathlon, para-sailing, para-shooting or para-swimming. | 9 years | 32 | 1 | Impairment related  Training load  Equipment  Competition/training phase |  | Non-significant reported differences in injury occurrence between various disability categories  Training load was reported most frequently related to injury occurrence  Equipment-related was reported related to injury occurrence  Competition was reported related to injury occurrence | 4 |
| **hirschmüller et al. 2021 [46]** | Injury and Illness Surveillance in Elite Para Athletes: An Urgent Need for Suitable Illness Prevention Strategies | Disorders of the musculoskeletal system as well as concussions were classified as injuries and further subcategorized into acute (onset linked to a specific injury event) and overuse injuries (no specific injury event) | Summer Paralympic sports | 29 weeks | 58 | 1, 2, 3 | Impairment related  Age  Weather/season | The mean prevalence of health problems varied between sex, with female and male athletes reporting of 33% and 24%, respectively  Based on the means of the first and final 5 weeks of data collection,  the prevalence of health problems in general reduced | No significant differences in IR of illnesses and injuries between paraplegic and nonparaplegic | 9 |
| **jarraya et al. 2021 [51]** | Sports injuries at the Rio de Janeiro 2016 Summer Paralympic Games: use of diagnostic imaging services | Sports-related bone, muscle, tendon, and ligament injuries in para athletes that underwent imaging. | Summer Paralympic sports | Rio 2016 summer Paralympic games | 4378 | 1, 2, 3 | Impairment related  Type of sports |  | Athletes with visual impairment had the highest proportion of imaging depicted injuries overall, followed by musculoskeletal, and neurologic impairment  Overall, most injuries were reported in athletics | 7 |
| **kasitinon et al. 2021 [54]** | Health-Related Incidents among Intercollegiate Wheelchair Basketball Players | Self-reported injury | Wheelchair basketball | 5,5 months | 28 | 1, 4 | Impairment related | Significant differences in risk ratios between wheelchair basketball players and NCAA basketball players. |  | 6 |
| **lexell et al. 2021 [56]** | Incidence of sports-related concussion in elite para athletes–a 52-week prospective study | an SRC was recorded: (i) if the athlete had a blow to or jolting of the head that had caused an acute disruption of brain function with concomitant symptoms; (ii) if it had caused changes in normal training or competition regarding the mode, duration, intensity, or frequency; and (iii) if it was confirmed by medical personnel. | Summer and Winter Paralympic sports | 52 weeks | 107 | 1, 2, 3 | Impairment related  Type of sports  Sex  Wheelchair use  Training load | Athletes with a vision impairment reported a significantly higher incidence proportion of SRC and a significantly higher incidence rate of SRC  Female athletes reported a significantly higher incidence rate of SRC compared to male athletes. | There were no significant relations regarding sport (summer vs winter and team vs individual)  There were no significant relations regarding athletes using a wheelchair or being ambulatory  There were no significant relations regarding training load (low, middle, high) | 9 |
| **maurice et al. 2021 [59]** | The 2019 Cameroon University Games: Prevention Strategies for Musculoskeletal Injuries | "Musculoskeletal injury was defined as any physical symptom that required medical attention, or prevented an athlete from taking full part in training and/or competition. These included all injuries that received medical attention, or caused performance restriction or time loss to the athletes’  training/competition and were newly incurred during the  Games." | Para-athletics, power-lifting and para-tennis | 2019 Cameroon University Games | 97 | NR | Impairment related |  | No reported injuries in Paralympic athletes while the prevalence in Olympic athletes was 10.4% | 8 |
| **steffen et al. 2021 [80]** | Illness and injury among Norwegian Para athletes over five consecutive Paralympic Summer and Winter Games cycles: prevailing high illness burden on the road from 2012 to 2020 | Any reported health problem, irrespective of its consequences on their sports participation or performance, and irrespective of whether they had sought medical attention | Summer and Winter Paralympic sports | 5 Paralympic cycles (winter and summer) | 94 | 1, 2, 3 | Impairment related  Type of sports  Sex | Athletes with neurological impairments reported more weekly illness, including substantial illness, and fewer injuries in general compared to athletes with musculoskeletal impairments  Female athletes reported a greater proportion of illnesses in general and fewer injuries compared to male athletes | The difference between winter versus summer sport athletes was minimal | 8 |
| NOTE: Disability type; 1) physically disabled 2) Visually disabled 3) Intellectual disabled 4) Mental health condition 5) Hearing impairment | | | | | | | | | | |
