# Supplementary material for: Sports-Related Health Problems in Para-Sports: A Systematic Review With Quality Assessment
Source: Sports Health. 2023 Jun 19;16(4):551–64. doi: 10.1177/19417381231178534 (PMC11195855; doi:10.1177/19417381231178534)
Supplement: sj-docx-5-sph-10.1177_19417381231178534 – Supplemental material for Sports-Related Health Problems in Para-Sports: A Systematic Review With Quality Assessment [file sj-docx-5-sph-10.1177_19417381231178534.docx]

*Appendix 5: Quality score assessment of the included studies reporting om the first two steps of the Sequence of Prevention model*

| **Study** | **(1) Definition of injury described in each study (yes/no)** | **(2) Study with prospective designs that presented incidence or prevelance data (yes/no)** | **(3) Description of the population of athletes (e.g. sport, disability, classification) or the player positions (e.g. goalkeepers or forward players) that participated in the study (yes/no)** | **(4) Was the process of inclusion of athletes in the study at random (i.e. not by convenience) or was the data collection performed with the entire target population (yes/no)** | **(5) Data analysis was performed with at least 80% of the athletes included in the study (yes/no)** | **(6) Were data regarding the injuries reported by a healthcare professional (yes/no)** | **(7) Was the same mode of data collection (e-mail, telephone, interview etc.) used (yes/no)** | **(8) Was the diagnosis conducted by medical team (yes/no)** | **(9) Follow-up period of at least 6 months for prospective studies (yes/no)** | **(10) Incidence or prevalence rates of injury expressed by a ratio that represents both the number of injuries as well as the exposure to sport (e.g. IR/1000 hours of sport exposure) (yes/no)** | **Total** |
| --- | --- | --- | --- | --- | --- | --- | --- | --- | --- | --- | --- |
| **Burham et al. 1991 [9]** | 0 | 0 | 1 | NR | 0 | 1 | 1 | 1 | 0 | 0 | 4 |
| **Burnham et al. 1993 [10]** | 0 | 0 | 1 | 0 | 1 | NR | 1 | NR | 0 | 0 | 3 |
| **Calmels et al. 1994 [12]** | 0 | 1 | 1 | 1 | NR | 1 | 1 | 1 | 0 | 0 | 6 |
| **Reynolds et al. 1994 [70]** | 0 | 1 | 1 | 1 | 1 | 1 | 1 | 1 | 0 | 0 | 7 |
| **Ferrara & Buckley. 1996 [33]** | 1 | 1 | 1 | 0 | NR | 0 | 1 | 1 | 1 | 1 | 7 |
| **Miyahara & Gerrard. 1997 [62]** | 0 | 0 | 1 | NR | 1 | 1 | 1 | 1 | 0 | 0 | 5 |
| **Curtis & Black. 1999 [16]** | 0 | 0 | 1 | NR | 0 | 0 | 1 | 0 | 0 | 0 | 2 |
| **Ferrara et al. 2000 [34]** | 1 | 1 | 1 | 1 | 1 | 1 | 1 | 1 | 0 | 0 | 8 |
| **Nyland et al. 2000 [64]** | 1 | 1 | 1 | 1 | 1 | 1 | 1 | 1 | 0 | 0 | 8 |
| **Fullerton et al. 2003 [37]** | 0 | 0 | 1 | 1 | 0 | 0 | 1 | 0 | 0 | 0 | 3 |
| **Finley & Rodgers. 2004 [36]** | 0 | 0 | 1 | 0 | NR | 0 | 1 | 1 | 0 | 0 | 3 |
| **Webborn et al. 2006 [90]** | 0 | 1 | 1 | 1 | 1 | 1 | NR | 1 | 0 | 0 | 6 |
| **Jeon et al. 2010 [52]** | 0 | 0 | 1 | NR | 1 | 0 | 1 | 1 | 0 | 0 | 4 |
| **Silva et al. 2011 [78]** | 1 | 1 | 1 | 1 | 1 | 1 | 1 | NR | 0 | 0 | 7 |
| **Chung et al. 2012 [13]** | 1 | 1 | 1 | NR | 1 | 1 | 1 | 1 | 1 | 1 | 9 |
| **Silva et al. 2013 [75]** | 1 | 1 | 1 | 0 | NR | 1 | 1 | 1 | 1 | 1 | 8 |
| **Webborn et al. 2012 [89]** | 1 | 1 | 1 | 1 | 1 | 1 | 1 | 1 | 0 | 1 | 9 |
| **Derman et al. 2013 [22]** | 1 | 1 | 0 | 1 | 1 | 1 | 1 | 1 | 0 | 1 | 8 |
| **Wilick et al. 2013 [93]** | 1 | 1 | 1 | 1 | 1 | 1 | 1 | 1 | 0 | 1 | 9 |
| **Silva et al. 2013 [77]** | 1 | 1 | 1 | NR | 1 | 1 | 1 | 1 | 0 | 1 | 8 |
| **Silva et al. 2013 [76]** | 1 | 1 | 1 | NR | 1 | 1 | 1 | 1 | 0 | 1 | 8 |
| **Gawronski et al. 2013 [40]** | 1 | 1 | 1 | 1 | 1 | 1 | 1 | 1 | 0 | 1 | 9 |
| **Derman et al. 2014 [21]** | 1 | 1 | 1 | 1 | 1 | 1 | 1 | 1 | 0 | 1 | 9 |
| **Mutsuzaki et al. 2014 [63]** | 0 | 0 | 1 | 1 | 1 | 1 | 1 | 1 | 0 | 0 | 6 |
| **Bauerfeind et al. 2015 [4]** | 1 | 1 | 1 | 1 | 0 | 1 | 1 | 1 | 1 | 1 | 9 |
| **Wilick et al. 2016 [92]** | 1 | 1 | 1 | 1 | 1 | 1 | 1 | 1 | 0 | 1 | 9 |
| **Webborn et al. 2016 [87]** | 1 | 1 | 1 | 1 | 1 | 1 | 1 | 1 | 0 | 1 | 9 |
| **Aytar et al. 2015 [2]** | 0 | 0 | 1 | NR | NR | 0 | 1 | 1 | 0 | 0 | 3 |
| **Derman et al. 2016 [25]** | 1 | 1 | 1 | 1 | 1 | 1 | 1 | 1 | 0 | 1 | 9 |
| **Derman et al. 2016 [24]** | 1 | 1 | 1 | 1 | 1 | 1 | 1 | 1 | 0 | 1 | 9 |
| **Blauwet et al. 2016 [5]** | 1 | 1 | 1 | 1 | 1 | 1 | 1 | 1 | 0 | 1 | 9 |
| **Tsunoda et al. 2016 [82]** | 0 | 0 | 1 | NR | NR | 0 | 1 | 0 | 0 | 0 | 2 |
| **Fagher et al. 2017 [32]** | 1 | 1 | 1 | 1 | 0 | 0 | 1 | 0 | 0 | 1 | 6 |
| **Shimizu et al. 2017 [74]** | 1 | 0 | 1 | 1 | 1 | 1 | 1 | 1 | 0 | 0 | 7 |
| **Kasinska & tasiemski. 2017 [53]** | 0 | 1 | 1 | NR | 1 | 1 | 1 | 1 | 1 | 1 | 8 |
| **Derman et al. 2018 [20]** | 1 | 1 | 1 | 1 | 1 | 1 | 1 | 1 | 0 | 1 | 9 |
| **Kubosch et al. 2017 [55]** | 1 | 1 | 1 | 1 | 1 | 1 | 1 | 1 | 0 | 1 | 9 |
| **Warner et al. 2018 [86]** | 0 | 0 | 1 | NR | NR | 0 | 1 | 0 | 0 | 0 | 2 |
| **Derman et al. 2018 [23]** | 1 | 1 | 1 | 1 | 1 | 1 | 1 | 1 | 0 | 1 | 9 |
| **Hollander et al. 2020 [48]** | 1 | 1 | 1 | 1 | 0 | 1 | 1 | 1 | 0 | 1 | 8 |
| **Ona Ayala et al. 2019 [65]** | 1 | 1 | 1 | 1 | 0 | 1 | 1 | 1 | 0 | 1 | 8 |
| **Pérez-tejro & Gómez. 2019 [68]** | 0 | 0 | 1 | NR | NR | 0 | 1 | 0 | 0 | 0 | 2 |
| **Ortega-Santiago et al. 2020 [66]** | 0 | 0 | 1 | 1 | NR | 0 | 1 | 0 | 0 | 0 | 3 |
| **Derman et al. 2020 [19]** | 1 | 1 | 1 | 1 | 1 | 1 | 1 | 1 | 0 | 1 | 9 |
| **cyr et al. 2020 [17]** | 1 | 1 | 1 | NR | NR | 1 | 1 | 1 | 0 | 0 | 6 |
| **fagher et al. 2020 [30]** | 1 | 1 | 1 | 1 | 1 | 0 | 1 | 0 | 1 | 1 | 8 |
| **heneghan et al. 2020 [43]** | 1 | 0 | 1 | NR | NR | 1 | 1 | 1 | 0 | 0 | 5 |
| **meirelles et al. 2020 [61]** | 1 | 0 | 1 | NR | 1 | 1 | 1 | 1 | 0 | 0 | 6 |
| **tamai et al. 2020 [81]** | 1 | 0 | 1 | 0 | 1 | 1 | 1 | 1 | 0 | 0 | 6 |
| **gutiérrez-santiago et al. 2020 [41]** | 1 | 1 | 1 | 1 | 1 | 0 | 1 | 0 | 0 | 1 | 7 |
| **busch et al. 2021 [11]** | 1 | 1 | 1 | NR | 1 | 0 | 1 | 1 | 1 | 1 | 8 |
| **brancaleone et al. 2021 [6]** | 0 | 1 | 1 | 0 | 1 | NR | 1 | NR | 1 | 0 | 5 |
| **heneghan et al. 2021 [44]** | 1 | 0 | 1 | NR | NR | 1 | 0 | 1 | 0 | 0 | 4 |
| **hirschmüller et al. 2021 [46]** | 1 | 1 | 1 | 1 | 1 | 0 | 1 | 1 | 1 | 1 | 9 |
| **jarraya et al. 2021 [51]** | 1 | 0 | 1 | 1 | 1 | 1 | 1 | 1 | 0 | 0 | 7 |
| **kasitinon et al. 2021 [54]** | 1 | 1 | 1 | 0 | 1 | 0 | 1 | NR | 0 | 1 | 6 |
| **lexell et al. 2021 [56]** | 1 | 1 | 1 | 1 | 1 | 0 | 1 | 1 | 1 | 1 | 9 |
| **maurice et al. 2021 [59]** | 1 | 1 | 1 | 1 | 1 | 1 | 1 | 1 | 0 | 0 | 8 |
| **santos et al. 2021 [73]** | 1 | 1 | 1 | 0 | 1 | 0 | 1 | NR | NR | 0 | 5 |
| **steffen et al. 2021 [80]** | 0 | 1 | 1 | 1 | 1 | 0 | 1 | 1 | 1 | 1 | 8 |
| **yamaguchi et al. 2021 [96]** | 0 | 1 | 1 | 1 | NR | 1 | 1 | 1 | 0 | 1 | 7 |
|  |  |  |  |  |  |  |  |  |  |  |  |
| **% “YES” INCLUDED STUDIES** | 67% | 69% | 98% | 59% | 67% | 66% | 97% | 77% | 18% | 52% |  |
| 10-point quality assessment tool by Hoy et al.[49] NOTE: 1: Yes, 0: No, NR: Not reported | | | | | | | | | | | |
