# Supplementary material for: Sports-Related Health Problems in Para-Sports: A Systematic Review With Quality Assessment
Source: Sports Health. 2023 Jun 19;16(4):551–64. doi: 10.1177/19417381231178534 (PMC11195855; doi:10.1177/19417381231178534)
Supplement: sj-docx-6-sph-10.1177_19417381231178534 – Supplemental material for Sports-Related Health Problems in Para-Sports: A Systematic Review With Quality Assessment [file sj-docx-6-sph-10.1177_19417381231178534.docx]

*Appendix 6: Risk of bias scores of the included trials using the Cochrane Risk of Bias assessment tool*

|  | **RANDOM SEQUENCE GENERATION**  **(Selection bias)** | **ALLOCATION CONCEALMENT**  **(Selection bias)** | **BLINDING OF PARTICIPANTS AND PERSONNEL**  **(Performance bias)** | **BLINDING OF OUTCOME ASSESSMENT**  **(Detection bias)** | **INCOMPLETE OUTCOME DATA**  **(Attrition bias)** | **SELECTIVE REPORTING**  **(Reporting bias)** | **OTHER BIAS**  **(Bias due to problems not covered elsewhere)** | **Total** |
| --- | --- | --- | --- | --- | --- | --- | --- | --- |
| **Wilroy et al. 2017 [94]** | 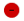 | 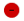 | 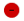 | 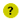 | 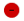 | 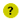 | 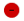 | 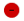 |
| **Garcia-Gomez et al. 2019 [39]** | 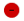 | 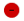 | 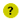 | 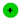 | 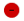 | 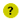 | 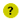 | 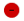 |
| **maarouf et al. 2021 [57]** | 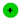 | 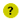 | 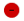 | 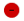 | 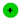 | 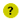 | 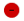 | 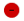 |
| note: 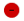-> high risk 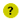-> unclear risk 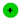-> low risk | | | | | | | | |
